# Microglial CX3CR1 signaling mediates stress-induced pain behavior in mice

**DOI:** 10.3389/fimmu.2026.1869876

**Published:** 2026-07-01

**Authors:** Barbara Fülöp, Ágnes Király, Rebeka Petrák, Júlia Müller, Tünde Biró-Sütő, Viktória Kormos, Valéria Tékus, Katalin Rozmer, Ádám Dénes, Éva Borbély, Zsuzsanna Helyes

**Affiliations:** 1 Department of Pharmacology and Pharmacotherapy, Medical School, University of Pécs, Centre of Neurosciences, Pécs, Hungary; 2 HUN-REN-PTE Chronic Pain Research Group, Pécs, Hungary; 3 National Laboratory of Translational Neuroscience, Budapest, Hungary; 4 Department of Pharmaceutical Chemistry, Faculty of Pharmacy, University of Pécs, Pécs, Hungary; 5 ”Momentum” Laboratory of Neuroimmunology, HUN-REN Institute of Experimental Medicine, Budapest, Hungary; 6 Eötvös Loránd Research Network, Chronic Pain Research Group, Pécs, Hungary; 7 National Drug Research and Development Laboratory, Budapest, Hungary

**Keywords:** chronic primary pain, chronic restraint stress, fibromyalgia, neuroinflammation, stress-induced pain

## Abstract

**Introduction:**

Chronic primary pain conditions, including fibromyalgia, affect up to 10% of the population, yet their pathophysiology is unexplored and the treatment is insufficient. Chronic stress is a key etiological factor and is known to modulate microglial function, partly via the CX3CR1 fractalkine receptor. Here, we investigated the role of CX3CR1 in a mouse model of stress-induced pain.

**Methods:**

Female and male CX3CR1-deficient (KO) and C57Bl/6J wild-type (WT) mice were exposed to chronic restraint stress (CRS) for 2 weeks. Mechanical and cold sensitivity were assessed before and during CRS. Microglia-IBA1 and astrocyte-GFAP activation were analyzed in stress- and pain-related brain regions, and neuron–glia interactions were examined in the somatosensory cortex hindlimb area (S1HL). Pharmacological validation was performed using the CX3CR1 antagonist, AZD8797 in WT mice.

**Results:**

In WT animals, CRS induced approximately 20% mechanical and 60-70% cold hyperalgesia. Mechanical pain and cold sensitivity was significantly reduced in stressed CX3CR1 KO mice of both sexes. CRS caused microglia and astrocyte integrated density increases in stress- and pain-related regions in WT but not CX3CR1 KO mice. Microglia coverage of neurons was greater in the S1HL region of KO animals independently of the CRS protocol. Pharmacological blockade of the CX3CR1 abolished CRS-evoked mechanical but not cold hyperalgesia.

**Discussion:**

These findings demonstrate that microglial CX3CR1 signaling contributes to chronic stress-induced pain through neuroinflammatory mechanisms and central pain sensitization. Targeting CX3CR1 may represent a promising therapeutic strategy for chronic primary pain conditions such as fibromyalgia.

## 1 Introduction

According to the definition, chronic primary pain is pain in one or more anatomical regions that persists or recurs for longer than 3 months. Furthermore, it is associated with significant emotional distress (e.g., anxiety, anger, frustration, or depressed mood) and/or functional disability (interference in activities of daily life and participation in social roles), and another diagnosis does not better account for the symptoms (1). Chronic psychosocial distress is an etiological and/or aggravating factor of several pain conditions, including fibromyalgia (FM) via systemic neuroendocrine dysregulations and neuroinflammation leading to central sensitization (2, 3). FM is a chronic primary pain syndrome characterized by widespread musculoskeletal pain, fatigue, mood disorders and cognitive disturbances (1, 4, 5) affecting more than 2% of the population (6, 7) with female predominance. Although the pathophysiological mechanisms are poorly understood, it is known that stress is the only identified causative factor (4, 8, 9). FM is still an unmet medical need since the traditional analgesics including nonsteroidal anti-inflammatory drugs and opioids, as well as adjuvant analgesics (certain antidepressants and antiepileptics) do not provide sufficient effect and/or have unfavorable side effect profiles (10–12). Therefore, considerable effort is invested in unraveling the complexity of the underlying molecular mechanisms and identifying novel drug targets.

A rapidly growing body of literature data on neuroinflammation is emerging, highlighting its pivotal role in FM pathogenesis. Both human data (13–15) obtained by the use of positron emission tomography (PET) radioligand tracer [11C] PBR28, referring to activated glia cells, and preclinical (16–18) experimental results demonstrate the importance of glial phenotype changes and glia-neuron interactions in pain persistence. These processes involve the alterations in microglial states through the C-X3-C motif chemokine receptor 1 (CX3CR1) (19, 20). In the periphery, the receptor is expressed on monocytes, dendritic cells, natural killer cells, and T cells; meanwhile, in the central nervous system (CNS), it is expressed exclusively on microglia cells, one of the most important immune cells of the brain and spinal cord (21). Fractalkine (CX3CL1) is the only known endogenous ligand of the receptor, binds to microglial CX3CR1, controlling the activation of several downstream signaling pathways, such as the activation of the intracellular p38 mitogen-activated protein kinase (MAPK) pathway that modulates the release of, among others, the proinflammatory cytokine interleukin-1 (IL-1) (22, 23). The CX3CL1–CX3CR1 interactions are key to modulate the crosstalk between neurons and microglia. This has been implicated in several neurodegenerative processes involved in Alzheimer’s and Parkinson’s diseases (24), as well as inflammatory mechanisms in the periphery related to atherosclerosis and arthritis (25). Although data support both the role of microglia in neuroinflammatory processes and neuroinflammation in the formation of FM, the direct link of microglial activation in FM, particularly regarding the function of CX3CR1, is still unclear.

Therefore, here we investigated the involvement of the CX3CR1, an important trigger of microglia activation, in a chronic stress-induced pain mouse model using both genetic deletion and pharmacological inhibition.

## 2 Materials and methods

### 2.1 Animals

Experiments were performed on 12-16-week-old female and male C57Bl/6J and CX3CR1 gene-deficient mice backcrossed for 7–9 generations to C57Bl/6J mice. C57BL/6J mice were used as wild-type (WT) controls alongside the genetically modified animals, and C57Bl/6J mice were used for experiments involving drug testing. The original C57Bl/6J breeding pairs were purchased from Charles River Ltd. (Wilmington, MA, USA).

The original breeding pairs of the CX3CR1 knock-out (KO) mice were generated at the HUN-REN Institute of Experimental Medicine as previously described (26).

All animals were bred and kept in the Laboratory Animal House of the Department of Pharmacology and Pharmacotherapy of the University of Pécs, maintained under a 12-h light-dark cycle (starting at 6:00 AM) at 22 ± 25 °C, 50% humidity, provided with standard mouse chow and water *ad libitum*. Mice were housed in groups of 2–6 in polycarbonate cages (330 cm^2^ floor space, 12 cm height) on wood shaving bedding. The health profile of the animals was regularly monitored according to the FELASA recommendations. All experiments were conducted in accordance with the ARRIVE guidelines.

### 2.2 The chronic restraint stress paradigm

The 2-week CRS paradigm was applied as published earlier (27). Briefly, the paradigm consisted of restraint for 6 hours daily for 2 weeks. Restriction tubes are 50-ml well-ventilated plastic tubes, in which the movements of the mice were restricted. Restraint stress routinely began in the morning. Non-stressed mice were identically handled, but during the time of the stress period, they were left in their home cages in the Laboratory Animal House (28).

### 2.3 Experimental design

Baseline nociceptive measurements were started 1 week before the two-week-long CRS protocol, as shown in **Supplementary Figure 1**. All initial nociceptive tests were performed three times on two non-consecutive days. Thereafter, the results were averaged and used as ‘Initial’ values. During the 2-week-long CRS, nociceptive tests were performed once a week. In contrast, behavioral tests were implemented only once, on the second week. Threshold measurements and behavioral tests were carried out in the laboratory of the Department at least 2 hours after the restraint stress had ended. The experimenter was always blind to the stress state of the animals. At the end of the two-week stress protocol, approximately 1 hour after the last measurement, the animals were anesthetized and perfused.

In total, KO and WT male and female mice were used across different experimental paradigms. Pain (mechanical and cold) sensitivity was assessed in 14–19 animals per group. Behavioral testing was performed just on a subset of animals, as the primary focus of the study was pain-related outcomes.

For immunohistochemical analyses, 6 paraformaldehyde-perfused brains per group were randomly selected. In experiments targeting the primary somatosensory cortex, sections from the same animals were processed for both DAB-based and fluorescent staining.

Additionally, in a separate cohort, we tested the drug candidate AZD8797, a selective inhibitor of the CX3CR1, using the same CRS paradigm. WT (C57Bl/6J) animals received intraperitoneal injections of AZD8797 in a 1 mg/kg dose or vehicle twice daily throughout the two-week protocol. The morning dose was administered prior to the onset of the restraint stress. The afternoon dose was given either after the daily measurements (when applicable) or, on days without testing, at least 2 hours after the completion of the restraint procedure. 7–11 animals per group were used, of which 6 per group were processed for immunohistochemical analyses. The AZD8797 stock solution was prepared as described earlier (29).

Exact sample sizes for each experiment and group are shown in the individual dot plots and figure legends within the Results section.

### 2.4 Nociceptive measurements

#### 2.4.1 Dynamic plantar aesthesiometry (DPA)

Mechanonociceptive thresholds of the hind paws were determined with a DPA (Ugo Basile, Italy) device. Mice were placed into plastic boxes resting on a metal mesh. After a 10-minute acclimatization period, the threshold was measured by increasing force on the plantar surface with a small-diameter, unsharpened metal needle (max. force: 10 g, ramp: 4 s). The electronic unit recorded the force at which the animals withdrew their paws due to pain, referred to as the mechanonociceptive threshold (30). Three values were assessed and averaged on both paws.

#### 2.4.2 Cold tolerance test

The hind paws’ cold tolerance was determined by measuring the withdrawal latencies from icy, 0 °C water (31). Mice were held gently, and the hind paws were separately immersed in ice-containing water at 0 °C. The time after which the animals withdrew their paws due to pain was recorded and referred to as the thermonociceptive threshold. Withdrawal latency time was recorded with a cut-off time of 180 sec.

### 2.5 Tests for assessing anxiety- and depression-like behavior

Anxiety and depression-like behavior were examined to exclude their confounding effect on nociceptive parameters. A possible sedative effect on spontaneous locomotor activity was also investigated in drug dosing experiments.

#### 2.5.1 Open field test (OFT)

The OFT is a sensorimotor test used to determine the exploration tendency (time spent in the peripheral area of the arena is proportional to anxiety) and the locomotor activity (moving) of rodents. At the beginning of the test, mice were placed in the center of a brightly lit open arena of 48*48*55 cm. During the 5-minute-long test, their behavior was recorded with a Noldus camera system and evaluated with the EthoVision^®^ XT version 11 (Noldus, Wageningen, Netherlands) video tracking software afterward. The time spent moving and the time spent in the periphery were assessed (32).

#### 2.5.2 Light–dark box test (LDB)

The box used during the test has a lit (white-painted, non-covered) and a dark (black-painted, roofed) compartment with a hole on the wall between them at the floor level. The lit compartment was illuminated at an intensity of approximately 1000 lux. In the LDB test, the natural light aversion of mice in the lit compartment competes with the curiosity to explore the novel environment. Anxiety level is directly proportional to the time spent in the dark compartment. Mice were placed into the light compartment at the beginning of the experiment, and their behavior was observed for 5 minutes, recorded with a Noldus camera system, and evaluated by the EthoVision^®^ XT version 11 (Noldus, Wageningen, Netherlands) video tracking software afterward. The time spent in the dark compartment was assessed (28).

#### 2.5.3 Tail suspension test (TST)

The original TST test has been used worldwide since 1986 to evaluate depression-like behavior in rodents. Mice were individually suspended by their tails with adhesive tape 50 cm above the surface. The ratio of escape-oriented movements (coping) and complete lack of movement (depressive-like behavior) was determined with the immobility time, measured in the last 4 minutes of the 6-minute-long test period (33).

#### 2.5.4 Forced swim test (FST)

In the FST, animals were placed individually in clear cylinders (height: 25 cm, diameter: 20 cm) containing 19 cm of water depth (22-24 °C). Like in the TST, mice react to an inescapable acute stress situation by alternating between struggling (swimming) and immobility (floating). The total duration of the stress exposure was 6 min, and the time of immobility (referring to the lack of escaping behavior) was measured during the final 4 min of the experiment (34).

### 2.6 Perfusion and tissue processing

Mice were intraperitoneally injected with an overdose of ketamine-xylazine solution (dose: 100 and 5 mg/kg). Following deep anesthesia (checked with toe pinch), the thoracic cavity was opened, and the animals were transcardially perfused with 0.1-mol/L phosphate-buffered saline (PBS) and then with 4% paraformaldehyde (PFA) solution. Next, their brains were dissected. The adrenal glands and thymuses were also removed, and their weights were measured. Following a one-day post-fixation in 4% PFA, 6 randomly selected mouse brains *per* group were sliced into 30-µm coronal sections with a vibratome (Leica, VT1000S). Five series of sections were prepared from each animal, were placed into PBS-azide solution and stored at 4 °C until further processing.

### 2.7 Immunohistochemistry

#### 2.7.1 Ionized calcium binding adapter protein 1 (IBA1)

IBA1 immunoreactivity (n=6/group) was revealed by the conventional avidin–biotin–immunoperoxidase protocol, described earlier (35). After washing in PBS, free-floating brain sections were incubated sequentially in (a) 1% hydrogen peroxide (H_2_O_2_) in PBS solution for 30 min, followed by a repeated wash with PBS; (b) citrate solution at 90 °C for 10 minutes, followed by a repeated wash with PBS; (c) PBS/0.5% Triton™ X100 at room temperature for 30 min; (d) 2% normal goat serum (Vector Laboratories, Burlingame, CA) in PBS at room temperature for 30 min; (e) 2% normal goat serum in PBS with rabbit anti-IBA1 (019-19741, Wako Chemicals GmbH, Neuss, Germany, 1:10.000 dilution) primary antibody at room temperature overnight, followed by 3X wash with PBS; (f) 1.5% biotinylated goat anti-rabbit IgG (Vector Labs) at room temperature for 60 min, after repeated PBS washing; (g) avidin–biotin complex at room temperature for 60 min, followed by a PBS wash. The resulting activity was developed in 3,3-diaminobenzidine (DAB, Sigma St. Louis, MO, USA) with 0.003% H_2_O_2_ solution. The sections were mounted onto gelatine-coated slides, dehydrated in alcohols, cleared with xylene, and coverslipped with DePex mounting medium (Fluka, Germany).

#### 2.7.2 Glial fibrillary acidic protein (GFAP)

GFAP immunoreactivity (n=6/group) was revealed using a conventional avidin–biotin immunoperoxidase protocol; for a detailed description, see the IBA1 section above. In the case of GFAP immunostaining, non-specific binding sites were blocked with 2% normal horse serum (Vector Laboratories, Burlingame, CA, USA) in PBS, and 2% normal horse serum in PBS with mouse monoclonal anti-GFAP (NCLLGFAP-GA5, Novocastra, 1:1.000 dilution) primary antibody was used. The secondary antibody was 1.5% biotinylated anti-mouse IgG (Vector Labs) (27).

### 2.8 Immunofluorescent staining

Frontal brain sections were made at the level of the primary somatosensory cortex representation of the hind limb (-0.46 to -0.7 mm caudal to bregma) to visualize microglia in conjunction with cortical neurons and further examine microglia-neuron interactions. Sections of the PAG (-3.8 to -4.24 mm caudal to bregma) were also used to study the interactions between microglia and astrocytes.

The staining procedure followed the same pre-treatment protocol as described for the DAB-based immunohistochemistry (section 2.7), including H_2_O_2_ incubation, heat-induced antigen retrieval, Triton X-100 permeabilization, and blocking steps.

For immunofluorescence, free-floating sections were incubated overnight at room temperature with a primary antibody solution in PBS containing 3% normal goat serum. The following primary antibodies were used: rabbit anti-IBA1 (019-19741, Wako Chemicals GmbH, 1:10.000), mouse anti-Kv2.1 (75-014, NeuroMab, 1:1.000), or mouse anti-GFAP (NCLLGFAP-GA5, Novocastra, 1:1.000). After PBS washes, sections were incubated for 3 hours at room temperature with Alexa Fluor-conjugated secondary antibodies: Alexa 647 goat anti-rabbit IgG (A32733, Invitrogen, 1:500) and Alexa 568 goat anti-mouse IgG (A11031, Invitrogen, 1:500). Nuclear counterstaining was performed with 4’,6-diamino-2-phenylindole (DAPI). Detailed staining procedures were performed as described earlier (36).

Sections were mounted on gold-coated glass slides and coverslipped using ProLong™ Gold Antifade Mountant (P36930, Invitrogen).

### 2.9 Microscopic imaging

#### 2.9.1 Avidin–biotin–immunoperoxidase stained samples

Analyses of cell numbers, cell activation, and integrated density measurements were performed with ImageJ 1.48 software on (37) micrographs of the areas of interest using an Olympus IX81 microscope equipped with an Olympus DP74 digital camera, using a 10× objective lens. The resolution of the acquired images was 1920 × 1080 pixels. All imaging parameters were identical for each image; three images/brain region/animal were assessed. Four pain-related brain areas were investigated: hippocampus cornu ammonis area 3 (CA3: -2.18 to -2.46 mm caudal to bregma); primary somatosensory cortex—representation of the hind limb (S1HL: -0.46 to -0.7 mm caudal to bregma); periaqueductal gray (PAG: -3.8 to -4.24 mm caudal to bregma); central amygdala (CeA: -1.0 to -1.7 mm caudal to bregma). The pictures were randomly assigned, and the investigator was blind to the experimental groups.

##### 2.9.1.1 Analysis

IBA1+ and GFAP+ cells were quantified in the region of interest (ROI) using unit area rectangle selection. The cell density of IBA1+ microglia and GFAP+ astrocytes (number of cell bodies visible/mm^2^) was counted in the selected ROIs.

As described earlier (38), a scoring system was used to investigate the activation degree of IBA1+ microglia and GFAP+ astrocytes. The scoring system considers the relative amounts of IBA1 and GFAP proteins based on morphological changes, ranging from 1 (for a resting state) to 5 (fully activated state).

To assess the Integrated Density (IntDen), the images were converted into a binary black-and-white format using the ImageJ processing tool. For counting the integrated density of IBA1- and GFAP-positive cells, 10×-magnification slides were used. Areas of interest within the mentioned brain areas were outlined, and an automated count of integrated density values was calculated for each image. The IntDen values are reported as arbitrary units (AU) (27).

#### 2.9.2 Immunofluorescent-stained samples

Confocal images were taken with Nikon Eclipse Ti2-E confocal microscope with 60x objective. Z-stack of the images (1024x1024 pixels) was taken over the region of interest in a range of 20 µm-s, with 0.5 µm interslice distance, and a pinhole size less than one Airy unit. Virtual colors were selected to depict fluorescent signals; blue for DAPI, green for IBA1 and red for Kv2.1 and GFAP. Digital images were adjusted for brightness and contrast using FIJI (version 1.53c, NIH, USA). Identical measuring frames were placed randomly along the surface of pyramidal cells.

##### 2.9.2.1 Analysis

Quantitative data analysis of each dataset was performed by at least two observers, blinded to the samples’ origin and the experiments.

We investigate the somatic microglial junctions, the direct interaction surface area between microglial processes and the cell bodies of cortical pyramidal neurons, as described earlier (36). For the analysis of somatic junction prevalence, confocal stacks with double immunofluorescent labeling (cell type-marker- neurons: Kv2.1, microglia: IBA1, and DAPI nuclear staining) were acquired from at least three different regions of the hind limb representation of the mouse cortex (S1HL). All labeled and identified cells were counted when the whole cell body was within the Z-stack. Neurons were considered to be contacted by microglia when a microglial process visibly touched them. Microglia cells were considered ‘satellites’ when close soma-to-soma contacts with neurons occurred (36).

We also examined the surface area of microglia-neuron interactions and their ratio. The acquired Z-stack images were manually analyzed in each 0.5 µm section across the entire stack. We reconstructed the neurons in a ‘three-dimensional’ manner and outlined them, then separately marked the microglia-neuron contacts in each layer. The neurons examined were divided into three groups: the ratio of satellite microglia (microglia cell bodies in contact with the neuron cell bodies), microglial processes contacting the neurons, and microglia cells not in contact with neurons (36).

### 2.10 Statistical analysis

The normal distribution of the data was checked by Shapiro–Wilk and Kolmogorov–Smirnov tests. In the case of datasets that did not represent a normal distribution, the Kruskal–Wallis test was applied, followed by Dunn’s test. Two-way analysis of variance (ANOVA), followed by Sidak’s test and two-way repeated measurement (RM ANOVA), followed by Tukey’s test, were preferred for datasets with a normal distribution. When comparing two groups, an unpaired two-tailed t-test was applied, depending on the experimental design. GraphPad Prism 8 software was used for statistical analysis. Data are presented as the mean ± standard error of the mean. Statistical results, including f values, and exact p-values for all tests, are summarized in **Supplementary Tables 1**–**3**, **6**.

### 2.11 Ethics

Hungary’s National Ethics Committee on Animal Research approved all procedures in 28th April 2023 (license No. BA02/2000-25/2023. issued by the Government Office of Baranya County, Hungary) and were performed according to the European legislation (Directive 2010/63/EU) and Hungarian Government regulation (40/2013., II. 14.) on protecting animals used for scientific purposes.

## 3 Results

### 3.1 CRS-induced mechanical and cold hyperalgesia are absent in CX3CR1 knock-out mice of both sexes

The mean baseline mechanonociceptive threshold of both WT and KO male mice was significantly higher (WT: 8.796 ± 0.3346, KO: 8.676 ± 0.2829) compared to the females (WT: 8.289 ± 0.3944; KO: 8.054 ± 0.3503) (WT p<0.0001; KO p<0.0001), while the cold sensitivity values were significantly lower in male WTs, but only tendentiously in KOs (WT: 162.1 ± 2.488; KO: 166.9 ± 2.121) compared to respective female group (WT: 171.2 ± 1.167; KO:171.4± 1.446) (WT p=0.0107; KO p=0.1762).

**Figure 1:**
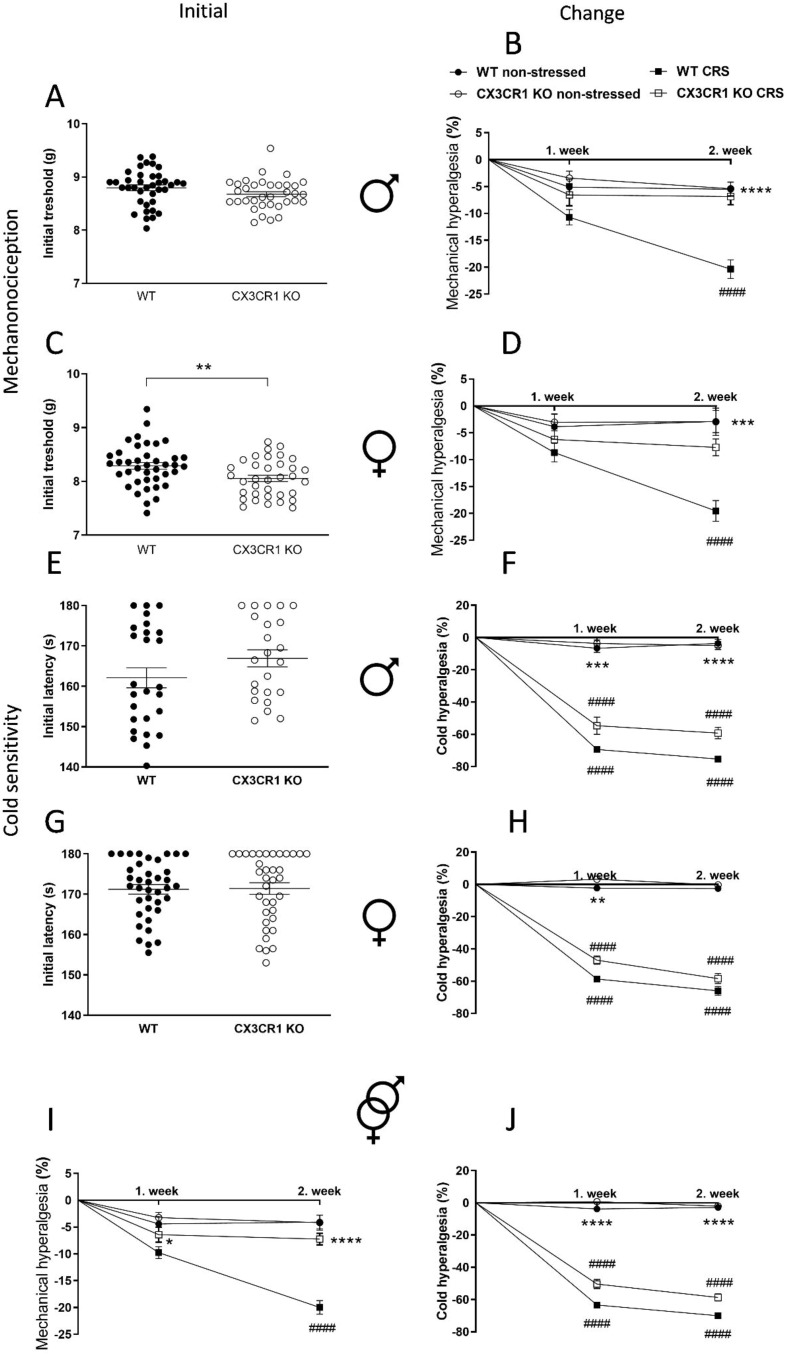
Baseline mechanonociceptive **(A, C)** and cold **(E, G)** thresholds of male and female wild-type (WT) and CX3CR1 knock-out (KO) mice. Data are presented as the mean ± SEM (n=23-40), animals with individual plots. Unpaired t-test, **p<0.01 vs. indicated groups. Effects of chronic restraint stress (CRS) on nociceptive behaviors of male **(B, F)**, female **(D, H)** animals, and both sexes together **(I, J)**. Mechanical and cold hyperalgesia are calculated by comparing the mechanonociceptive thresholds and withdrawal latencies to their self-control baseline values. Data are presented as the means ± SEM of n=14–21 animals; two-way repeated measurement analysis of variance (ANOVA), followed by Sidak’s tests; *p<0.05, **p<0.01, ***p<0.001, ****p<0.0001 vs. respective WT group; ^####^p< 0.0001 vs. respective non-stressed group.

Baseline mechanonociceptive thresholds of CX3CR1 KO mice were significantly lower than their WT counterparts in the female but not in the male group (**Figures 1A, C**). No differences were found between WT and CX3CR1-deficient animals in either sex regarding cold tolerance (**Figures 1E, G**). Both male and female WT mice developed approximately a 20% decrease in the mechano-nociceptive threshold (mechanical hyperalgesia) by the second week (**Figures 1B, D**) and around a 60% decrease in cold sensitivity beginning from the first week in response to CRS (**Figures 1F, H**). In contrast, CX3CR1 KO animals of both sexes did not develop stress-induced mechanical hyperalgesia (**Figures 1B, D**). Cold hyperalgesia was significantly lower during the whole experimental period in male, but only after the first week in female CX3CR1 KO compared to their WTs (**Figures 1F, H**). Analysis of the pooled male and female datasets revealed similar trends to those observed in the sex-specific analyses (**Figures 1I, J**).

### 3.2 Behavior tests

In stressed animals, the time spent immobile was significantly reduced for both genotypes in the TST, but there was no stress-induced change in immobility time in the FST. The OFT showed a genotype difference; the KO animals tended to spend less time in the periphery and moved less regardless of the stress application. Stress caused an elevated time spent moving in both genotypes, but the distance moved was elevated significantly only in the KOs. No difference was detected between the groups during the LDB test (**Supplementary Figure 2**).

### 3.3 Changes in body weight, thymus, and adrenal weight following CRS

At the baseline point, the age-matched CX3CR1 KO animals were significantly smaller than the WT mice in both sexes (WT: ♂: 27.59 ± 0.3878; ♀: 20.70 ± 0.1726; KO: ♂: 26.36 ± 0.4287; ♀: 19.43 ± 0.3614) (WT p<0.0001; KO p<0.0001). The CRS protocol caused a similar weight drop in both genotypes from the first week.

Relative thymus weights of female animals were significantly higher in both genotypes (WT: p=<0.0001 ♂: 0.001183 ± 0.0001097 mg/g; ♀: 0.002079 ± 6.289e-005 mg/g; KO: p=<0.0001 ♂: 0.001267 ± 8.168e-005 mg/g; ♀: 0.002387 ± 0.0001891 mg/g) (WT p<0.0001; KO p<0.0001). Thymus weight was significantly elevated after the stress protocol in both males and females in the two investigated genotypes.

Relative adrenal weights of female animals were significantly higher in both genotypes (WT: p=0.0014 ♂: 0.0001433 ± 1.531e-005 mg/g; ♀: 0.0002427 ± 1.604e-005 mg/g; KO: p=<0.0001 ♂: 0.0001769 ± 1.872e-005 mg/g; ♀: 0.0003179 ± 2.981e-005 mg/g) (WT p=0.014; KO p<0.0001). The same tendency of relative adrenal gland weight-gain was observed in both genotypes and both sexes due to CRS (**Supplementary Figure 3**).

### 3.4 CRS-induced microglial priming in the somatosensory cortex of WT but not in CX3CR1 KO mice

**Figure 2:**
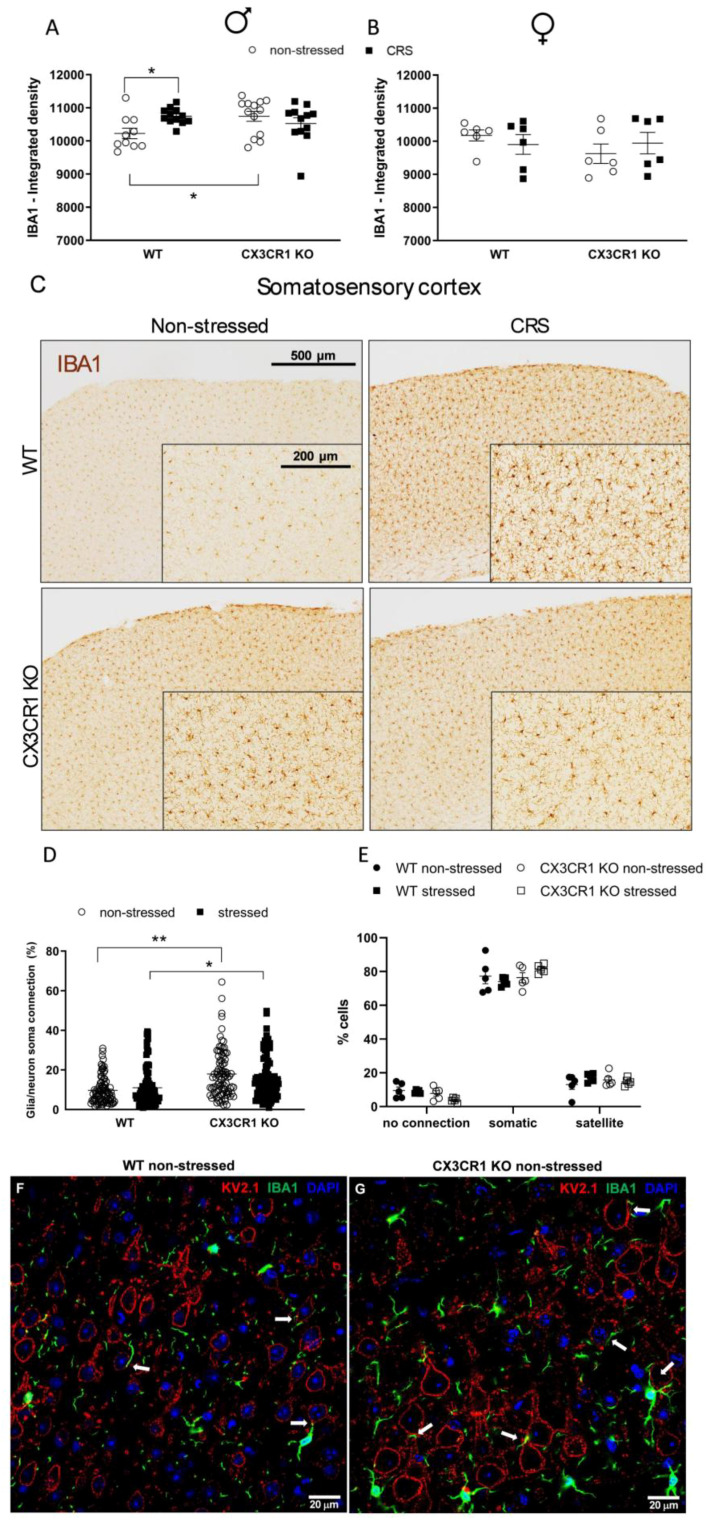
Effects of chronic restraint stress (CRS) on ionized calcium binding adapter protein 1 (IBA1) integrated density in the somatosensory cortex (S1HL) of male [**(A)**, n=11-13/group] and female [**(B)**, n=6/group] wild-type (WT) and CX3CR1 knock-out (KO) mice. Data are presented as the mean ± SEM, animals with individual plots. Two-way analysis of variance (ANOVA), followed by Tukey’s tests; *p<0.05. Representative images show the IBA1+ microglia cells in male animals’ S1HL **(C)**. #Effects of CRS on contact prevalence between microglia and neurons in WT and KO mice [**(D)**, n=18/neurons from 5 mice] and percentage of non-connecting, microglia with somatic contacts and satellite microglia cells [(E), n=5]. Data are presented as the mean ± SEM, animals with individual plots. Two-way analysis of variance (ANOVA), followed by Sidak’s tests; *p<0.01, **p<0.0001 vs. indicated groups. Representative images of neuron-glia connections in control WT and CX3CR1 KO male animal somatosensory cortex. White arrows indicate microglial processes (green) contacting Kv2.1 clusters (red) on neuronal somas in perfusion-fixed brain tissue samples of the somatosensory cortex in WT **(F)** and CX3CR1 KO **(G)** animals.

The baseline IBA1 integrated density values referring to microglial phenotype changes and morphological transformation (size and arborization) in the hind paw representation area of the somatosensory cortex (S1HL) did not show sex difference in WT mice, but in KO animals, males’ results were significantly higher (♂: 10743 ± 150.7; ♀: 9625 ± 292.0) (WT p=0.9785; KO p=0.0005), and significantly higher in male but not in female CX3CR1 KOmice compared to their WTs (**Figures 2A, C**). Significant CRS-induced microglia integrated density elevation in S1HL developed in male WT but not in CX3CR1 KO mice, interestingly, this elevation was not observed in females (**Figure 2B**).

### 3.5 Elevated microglial process coverage of Kv2.1 positive neuronal cell bodies in CX3CR1 KO mice

Microglia-neuron interactions determined by IBA1 and Kv2.1 immunofluorescent signal quantification suggest increased microglial process coverage of neurons in both non-stressed and stressed CX3CR1 KO mice compared to their respective WTs, but these values were not affected by CRS (**Figure 2D**). The same proportions of unconnected, microglia with somatic contacts, and satellite microglia were present in all groups, independent of genotype and stress (**Figure 2E**).

### 3.6 Higher stress-induced integrated IBA1 density values in male WT animals in the PAG but not in other investigated brain regions

In the PAG region, non-stressed female animals of both genotypes exhibited significantly higher integrated density values compared to male animals of the same genotype (WT: ♂: 7422 ± 159.6; ♀: 10526 ± 228.5; KO: ♂: 8800 ± 245.2; ♀: 10522 ± 221.6) (WT and KO p<0.0001). No significant difference was observed between non-stressed male and female animals integrated density values within the CA3 region (WT: ♂: 9667 ± 166.7; ♀: 9552 ± 296.3; KO: ♂: 9607 ± 429.1; ♀: 9325 ± 258.4) (WT p=0.9566; KO p=0.7663), neither in the CeA region (WT: ♂: 8882 ± 502.1; ♀: 9945 ± 333.4; KO: ♂: 9441 ± 403.8; ♀: 9627 ± 283.9) (WT p=0,1312; KO p=0.9319).

**Figure 3:**
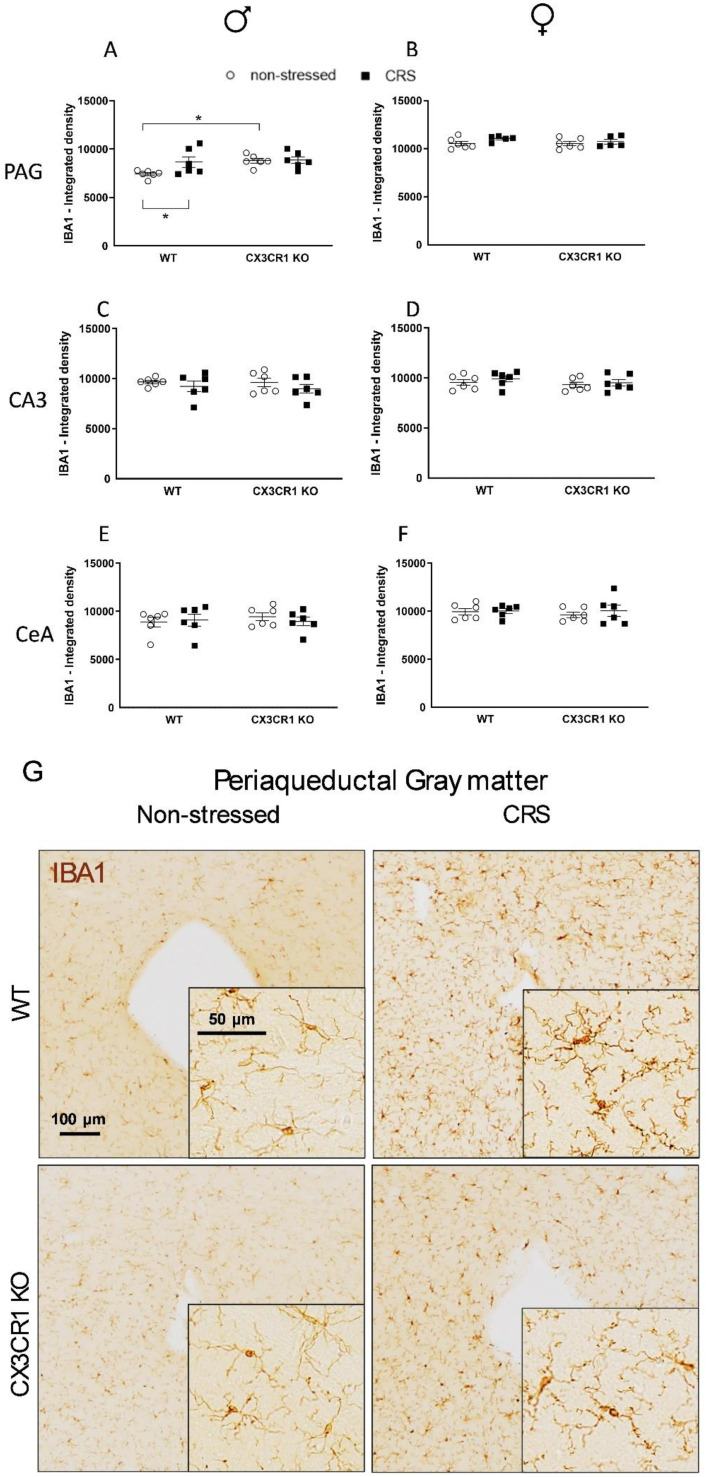
Effects of chronic restraint stress (CRS) on ionized calcium binding adapter protein 1 (IBA1) integrated density in different stress- and pain-related brain regions of male and female wild-type (WT) and CX3CR1 knock-out (KO) mice; in the periaqueductal gray matter [PAG; **(A, B)**], hippocampus Cornu Ammonis 3 [CA3; **(C, D)**] and central amygdala [CeA; **(E, F)**]. Data are presented as the mean ± SEM, animals with individual plots (n=6/group). Two-way analysis of variance (ANOVA), followed by Tukey’s tests; *p<0.05, vs. indicated groups. Representative images show the IBA1+ microglia cells in PAG in male animals’ samples **(G)**.

CRS induced a significant elevation in the IBA1 integrated density in the PAG area in males, but not in females. In this area, non-stressed male KO animals showed significantly higher levels of integrated density compared to WT animals (**Figures 3A, B, G**). In other examined brain regions, the four groups showed no significant difference in IBA1 integrated density in either sex (**Figures 3C–F**).

### 3.7 CRS-induced integrated GFAP density elevation is absent in CX3CR1 animals

Baseline sex differences in GFAP integrated density were observed among the non-stressed groups. In the PAG, significantly higher GFAP integrated density values were detected in non-stressed KO females compared to KO males, while no sex-related differences were found in the WT group (WT: ♂: 8569 ± 204.5; ♀: 7021 ± 805.3; KO: ♂: 8782 ± 569.7; ♀: 11124 ± 303.6) (WT p=0.0985; KO p=0.0101). In the CA3 region, WT non-stressed males exhibited significantly higher values than WT females, whereas no sex-related differences were found in the KO animals (WT: ♂: 8450 ± 349.3; ♀: 4983 ± 318.3; KO: ♂: 7979 ± 335.8; ♀: 6300 ± 810.4) (WT p=0.0002; KO p=0.0572).

**Figure 4:**
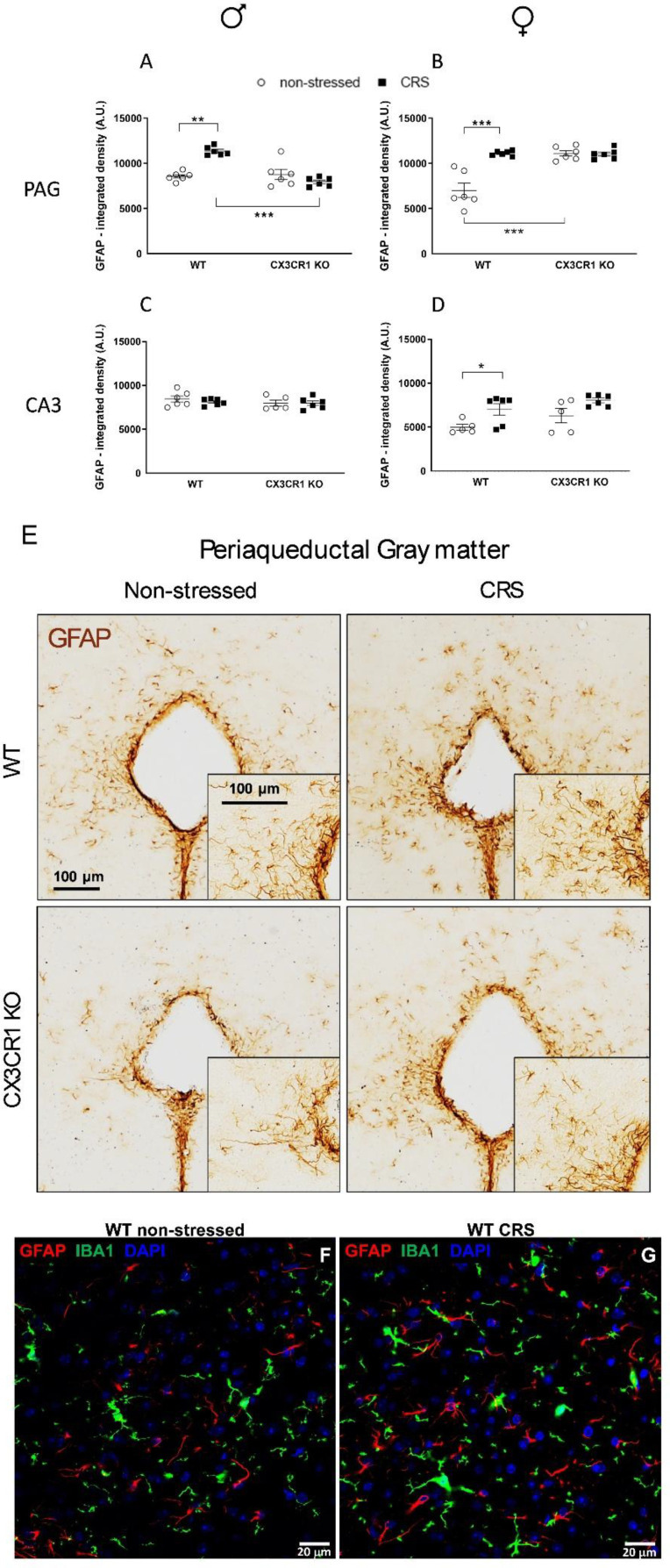
Effects of chronic restraint stress (CRS) on glial fibrillary acidic protein (GFAP) integrated density in different stress- and pain-related brain regions of male and female wild-type (WT) and CX3CR1 knock-out (KO) mice; in the periaqueductal gray matter (PAG) **(A, B)** and hippocampus Cornu Ammonis 3 (CA3) **(C, D)**. Data are presented as mean ± SEM, animals with individual plots (n=6/group). Two-way analysis of variance (ANOVA), followed by Tukey’s tests; *p<0.05, **p<0.001, ***p<0.0001 vs. indicated groups. Representative images show the GFAP+ astrocyte cells in PAG in male mice **(E)** Representative images of astrocyte-microglia interactions of the PAG in control and stressed WT male animals. Confocal images show microglia- and (green) astrocyte processes (red) contacting in the PAG of control **(F)** and after CRS **(G)** WT animals.

In both sexes, the GFAP integrated density in the PAG region was elevated in the WT animals following CRS (**Figures 4A, B, F, G**), and it was higher compared to the male stressed KO animals (**Figure 4A, E**). In female animals, the baseline integrated density was higher in the mice lacking CX3CR1 than in WT (**Figure 4B**). While the GFAP integrated density of the CA3 brain area in males was the same between the four groups (**Figure 4C**), in females, there was a significant increase in stressed WT animals but not in CX3CR1 KO ones (**Figure 4D**).

### 3.8 Microglia activation score elevation in WTs following CRS and *ab ovo* altered microglial density in KOs in stress and pain-related brain regions

There was no significant sex difference in cell density or activation in the examined regions in non-stressed animals (S1HL, PAG, CA3, CeA) (**Supplementary Table 4**).

Significantly elevated activation scores were observed in stressed WT animals in both sexes in the hippocampus CA3 region, and in the somatosensory cortex-hind limb representation of males, which elevation was not present in the KO animals. No change was present in the case of the CeA and in the PAG in either sex regarding the activation score.

In case of cell density, in males, significant elevation was present in both genotypes in the somatosensory cortex-hind limb representation region following CRS. Activation score elevation following CRS was present in the WTs, but not in KO animals, in the CeA region in males. We found an *ab ovo* altered, lower microglia cell density in the PAG in male and the somatosensory cortex and CeA in female CX3CR1 KO animals (**Supplementary Figure 4**).

### 3.9 Elevated astrocyte cell activation in WTs following CRS in CA3 and PAG in female but not male animals

There was no significant sex difference in astrocyte cell density or activation in the examined regions (PAG, CA3) (**Supplementary Table 5**).

In female animals, astrocyte activation was significantly increased in WT animals after CRS, in the hippocampus CA3 region, but not in the PAG. No differences in these parameters were found between the four groups in the investigated brain areas in male animals (**Supplementary Figure 5**).

### 3.10 CX3CR1 antagonist AZD8797 alleviates stress-induced mechanical but not cold hyperalgesia in male mice

**Figure 5:**
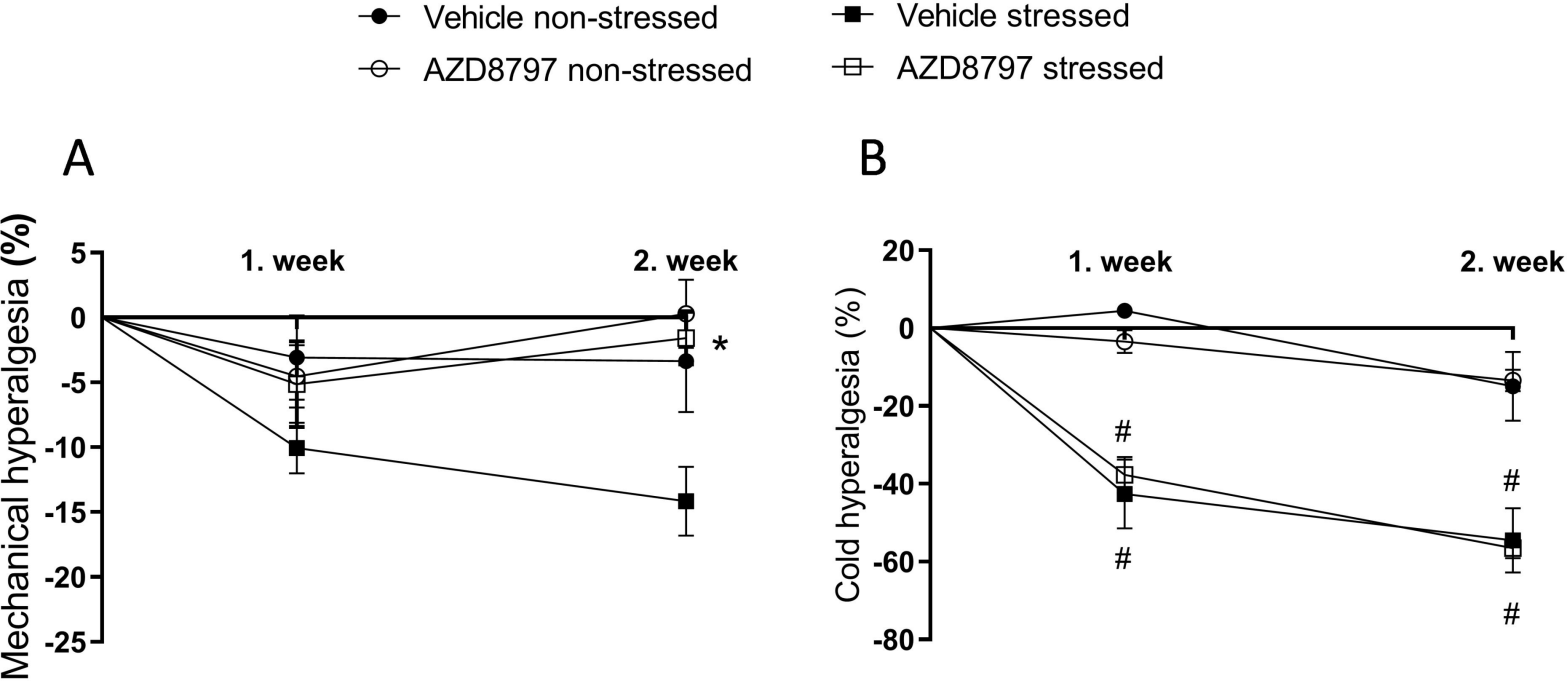
Effects of chronic restraint stress (CRS) on nociceptive behaviors of wild-type (WT) and CX3CR1 knock-out (KO) male mice. Mechanonociceptive threshold **(A)** and cold tolerance [paw withdrawal latency, **(B)**] changes are presented compared to the baseline threshold. Data are presented as the mean ± SEM (n= 7–11); two-way repeated measurement analysis of variance (ANOVA), followed by Sidak’s tests; *p<0.05 vs. respective vehicle-treated group; ^#^p<0.0001 vs. respective non-stressed groups.

Male animals treated with vehicles showed a significant decrease in the mechanonociceptive threshold due to stress, which was abolished by administration of the CX3CR1 antagonist compound, AZD8797 (**Figure 5A**). The cold hyperalgesia induced by the CRS protocol was to the same extent in vehicles and AZD8797-treated groups (**Figure 5B**).

### 3.11 AZD8797 administration altered OFT test results regardless of the stress

The time spent immobile was significantly lower in the FST for the stressed mice receiving the vehicle, but not for those receiving AZD8797 treatment. No significant difference was found between the groups regarding the TST. The non-stressed animals receiving AZD8797 spent significantly less time in the periphery and more time moving than the animals receiving the vehicle. No such difference was observed between the stressed animals receiving different treatments. No difference was found between the groups during the LDB test (**Supplementary Figure 6**).

### 3.12 No microglia or astrocyte alteration in stress-related brain regions, regardless of the pharmacological treatment

In either group, no changes in microglia and astrocyte cell parameters (integrated density, cell density, and cell activation) were observed (**Supplementary Figure 7**).

## 4 Discussion

We provide here the first data that microglial CX3CR1 contributes to chronic stress-induced pain behaviors, possibly via modulating glia-neuron interactions in the PAG and S1HL, the major pain-processing areas. Besides directly modulating microglia, CX3CR1 also triggers astrocyte activation in the PAG. The higher IBA1 integrated density in the PAG and S1HL of non-stressed male mice suggests that CX3CR1 also contributes to baseline microglia activity. Furthermore, it is demonstrated that blocking the CX3CR1 receptor by a selective antagonist provides novel perspectives for the treatment of stress-induced pain conditions, including FM.

In agreement with the literature data, baseline measurements in both genotypes revealed that female mice exhibited greater susceptibility to mechanical stimuli and lower sensitivity to cold, accompanied by lower body weights and higher relative thymus and adrenal gland weights compared to males (39–41). The greater IBA1 integrated density in the S1HL region of male CX3CR1 KO animals compared to females, and in the PAG of female animals of both genotypes compared to males, can be attributed to the established sex-dependent structural and functional differences in rodent microglia (42, 43). Moreover, the higher integrated GFAP density in the CA3 region of male mice and the PAG of females may also be explained by similar sex-dependent variations in the morphology and function of astrocytes, as reported in the literature (44, 45).

There is increasing evidence that chronic stress plays a crucial role in both the onset and progression of FM (1, 46, 47). Our data showed that stress-induced mechanical and cold hyperalgesia similarly developed in both sexes, despite clinical data that one and a half times more women suffer from FM compared to men (6). However, some studies suggest that FM is underdiagnosed in men, rather than being less common (48–50). Emerging literature suggests that chronic stress can induce neuroinflammation. Different types of chronic stress in preclinical studies increase the release of pro-inflammatory cytokines from microglia in brain regions involved in both stress and pain regulation, such as the hippocampus (18, 51–53). Neuroinflammation was demonstrated with translocator protein ligands expressed by activated glia cells in PET, including the primary somatosensory and motor cortex of FM patients (13, 14). Choline levels linked to glial activation were also elevated in insular and putamen regions of FM patients, positively correlating with pain intensity, determined by functional magnetic resonance imaging (15). In rats, neuroinflammation was described in reserpine- and acid saline-induced FM models, with elevated GFAP and IBA1 immunostaining intensities in the lumbar spinal dorsal horn (54–57). We found CRS-induced IBA1 integrated density elevation in the S1HL and PAG regions only in male WT mice. Integrated density analysis is a quantitative method combining the extent and intensity of immunohistochemical staining. It is an unbiased measure of overall marker expression, facilitating objective quantification of microglia and astrocyte cell responses while reducing the subjectivity inherent in morphology-based cell classification. Activation scores and cell densities were also determined; the integrated density provided the most appropriate and reproducible parameter to assess microglia activation (58). Literature data support that CRS induced a significant CX3CL1 mRNA expression increase, referring to neuroinflammation in the CA3 and orbitofrontal region of male, but not female rats. Moreover, a dramatic reduction in microglia activation in the prefrontal cortex was shown after CRS only in female rats, but not in males (59). Total microglia density in the hippocampus was not affected by stress application, but CRS altered the proportion of activated microglia cells, which change was present only in male animals (60).

Our results showed stress-induced GFAP increase in the PAG of both sexes, while in the CA3 region, this was observed only in females. Broad literature demonstrated that astrocytes have protective roles in neuronal homeostasis under normal conditions, but noxious stimuli can activate them in a sex-dependent manner, leading to pathological functions in several neurological diseases (61). This activation process contributed to pro-inflammatory cytokines like tumor necrosis factor alpha and IL-1 released from microglia (62).

In agreement with our earlier and literature data, immobility in the FST was reduced after two weeks of stress exposure in both sexes and genotypes. These data suggest that after chronic stress application, immobility does not necessarily reflect depression-like behavior like in the acute test, but anxiety and increased locomotion are likely to modify these outcome parameters depending on the type and duration of stressors (63, 64). This is supported by the findings that in the OFT, time spent moving also increased in response to CRS (65, 66). Other depression and anxiety-like behaviors in the TST and LDB did not show stress-induced alterations after two weeks.

CX3CL1 is cleaved from damaged or malfunctioning neurons and is the only known endogenous agonist of CX3CR1, which is exclusively expressed on microglial cells in the brain parenchyma. Besides, a subset of border-associated macrophages express CX3CR1 in the brain perivascularly and in the meninges and it is also expressed in different immune cells such as NK cells, T cells and monocytes/macrophages in the periphery (21, 67–73). Microglia in the cortex, striatum and hippocampus, but not the retina and thalamocortical regions of mice showed reduced ramification, referring to reduced activity in CX3CR1 KO mice (74–77). Aberrant activation and phagocytic ability of CX3CR1 KO microglial cells were reported (78, 79), which data coincides with the baseline significantly higher IBA1 integrated density difference we detected in CX3CR1 KO animals compared to WTs. The role of CX3CR1 has been shown in neuroinflammation related to multiple sclerosis (29), depression (80) and Alzheimer’s disease (78). Broad literature data demonstrate that CX3CR1 KO mice, having microglia deficiencies, are more resistant to different types of stressors and less likely to develop depression and anxiety-like behaviors (81–85). Here, we describe for the first time that CX3CR1 deficiency attenuates stress-induced pain parameters in mice.

Limited data are available regarding the involvement of CX3CR1 in pain, but elevated CX3CL1 levels were found in the corticospinal fluid of FM patients (86). According to preclinical data, CX3CR1 has been demonstrated to have a role in neuropathic pain (87). Formalin-induced acute inflammatory and partial sciatic nerve ligation-induced chronic neuropathic pain behaviors were reduced in CX3CR1 knockout mice (88, 89). Both thermal and mechanical sensitization were triggered in rats by intrathecal CX3CL1 injection (90). Furthermore, a CX3CL1 antibody reduced neuropathic pain in rats (91).

Although the specific mechanisms of microglial activation during stress response are not fully understood, the CX3CL1-CX3CR1 pathway seems to be a key component (92). CX3CR1 deficiency in mice prevents chronic unpredictable stress-related microglial arborization area and neuronal plasticity in the hippocampus CA1 (93). CRS increased IBA1 integrated density in the S1HL and PAG in WT, but not in CX3CR1 KO mice. Although in the S1HL, the proportion of direct contacts, somatic contacts, and satellite contacts between microglia and cortical neurons were affected neither by the genotype nor the stress itself, baseline neuron and microglia process coverage of neuronal somata was significantly greater in mice lacking CX3CR1. Somatic purinergic junctions have been identified as a major form of microglia-neuron interaction that is apparent across all brain areas tested in both rodents and in the human brain (36, 94–96). While somatic Kv2.1 clusters contacted by microglia have been identified as a marker for somatic purinergic junctions, Kv2.1 is expressed primarily by excitatory neurons in the cerebral cortex and thus the analysis performed may not be representative for inhibitory neurons in the present paper. In this paper we focused largely on somatic purinergic junctions as a major form of interaction between microglial processes and neuronal cell bodies. However, microglia establish a broad spectrum of interactions with neurons including those with synapses, dendrites or axon initial segments, not mentioning indirect interactions via astrocytes, oligodendrocytes or other cells. Therefore, the analysis performed in the paper may not be representative for all the different types of microglia-neuron interactions and effects at other sites might have remained unnoticed. CX3CR1 KO mice exhibit reduced dendritic spine pruning, abnormal synapse maturation, decreased functional connectivity between multiple cortical regions, as assessed by resting-state functional MRI, reflecting impaired synchronization of spontaneous neural activity (71, 97). Microglia-neuron interactions are considered bidirectional, in which neurons are suspected to regulate microglial purinergic signaling via CX3CL1–CX3CR1 signaling, and microglia, in turn, modulate neuron excitability and plasticity (98–101). CX3CR1 is also essential in neuronal development and appropriate neuronal connectivity (97, 102). Based on the present data, microglia cells lacking CX3CR1 may compensate for the functional defects by more extensive neuronal coverage.

CX3CR1 deficiency also abrogated the integrated GFAP density increase related to astrogliosis in the PAG and hippocampus in response to CRS, supporting CX3CR1-dependent microglia-astrocyte interactions. This finding is supported by reduced astrogliosis in CX3CR1 KO mice in an Alzheimer’s disease model (79), as well as *in vitro* data showing proliferative effect and increased IL-6 production of activated microglia on astrocytes (103). These results with KO mice suggest a potential for CX3CR1 as a drug target for chronic stress-induced pain.

The CX3CR1 antagonist AZD8797, which is currently in Phase II clinical trials as an immunomodulator for the treatment of women with estrogen-dependent cancers (104), abolished chronic stress-induced mechanical hyperalgesia in our model, due to both central and peripheral sensitization mechanisms. In contrast, AZD8797 did not influence mainly peripheral sensitization-induced cold hyperalgesia (105). This finding is supported by the concept that stress-induced mechanical hyperalgesia involves complex microglia-neuron interactions via CX3CR1 activation. However, it is presently unclear to what extent peripherally administered AZD8797 may block CX3CR1 in parenchymal microglia. Based on the currently available literature, AZD8797 appears to have limited blood–brain barrier penetration under physiological conditions (29, 106). However, chronic stress is known to impair blood–brain barrier integrity and increase its permeability, which may facilitate central access of compounds that otherwise show restricted penetration (107, 108). In addition, stress-induced hyperalgesia involves complex neuroimmune interactions between peripheral and central mechanisms. Therefore, peripheral modulation of CX3CR1-dependent inflammatory pathways may also indirectly influence central sensitization and pain processing (3, 109, 110). These suggest that the effects of AZD8797 may involve both central and peripheral mechanisms.

AZD8797 has been extensively studied in different animal models. Its anti-inflammatory effects improved cardiac hypertrophy in mice (111), prevented migraine-like hyperalgesia and thalamic and microglial activation in a status epilepticus rat model (100). Furthermore, CX3CL1 infusion exacerbated hyperalgesia in the periorbital region and microgliosis in the thalamus (112). However, AZD8797 alone or in combination with sub-analgesic morphine doses did not alter acute nocifensive behaviors in the cold-water tail flick test in rats and in the formalin test in mice (113).

A limitation of our study is the use of developmental CX3CR1 KO mice, in which compensatory mechanisms and the involvement of its non-microglial expression cannot be ruled out (114).

## 5 Conclusion

It is concluded that microglial CX3CR1 contributes to chronic stress-induced pain in mice, potentially involving altered glia–neuron interactions and related central sensitization mechanisms. CX3CR1 might be a promising drug target for the treatment of chronic primary pain conditions related to stress, such as FM. The CX3CR1 antagonist, AZD8797, already under clinical trials for other indications, would be worth testing in such a patient population.

## Data Availability

The original contributions presented in the study are included in the article/**Supplementary Material**. Further inquiries can be directed to the corresponding authors.

## References

[1] Nicholas M Vlaeyen JWS Rief W Barke A Aziz Q Benoliel R . The IASP classification of chronic pain for ICD-11: chronic primary pain. Pain. (2019) 160:28–37. doi: 10.1097/j.pain.0000000000001390 30586068

[2] Findeisen K Guymer E Littlejohn G . Neuroinflammatory and immunological aspects of fibromyalgia. Brain Sci. (2025) 15:206. doi: 10.3390/brainsci15020206 40002538 PMC11852494

[3] Ji RR Nackley A Huh Y Terrando N Maixner W . Neuroinflammation and central sensitization in chronic and widespread pain. Anesthesiology. (2018) 129:343. doi: 10.1097/aln.0000000000002130 29462012 PMC6051899

[4] Galvez-Sánchez CM Duschek S Del Paso GAR . Psychological impact of fibromyalgia: current perspectives. Psychol Res Behav Manag. (2019) 12:117–27. doi: 10.2147/PRBM.S178240 PMC6386210 30858740

[5] Seto A Han X Price LL Harvey WF Bannuru RR Wang C . The role of personality in patients with fibromyalgia. Clin Rheumatol. (2019) 38:149–57. doi: 10.1007/s10067-018-4316-7 30276562 PMC6364301

[6] Queiroz LP . Worldwide epidemiology of fibromyalgia topical collection on fibromyalgia. Curr Pain Headache Rep. (2013) 17(8):356. doi: 10.1007/s11916-013-0356-5 23801009

[7] Marques AP Santo ASE Berssaneti AA Matsutani LA Yuan SLK . Prevalence of fibromyalgia: literature review update. Rev Bras Reumatol (English Ed). (2017) 57:356–63. doi: 10.1016/j.rbre.2017.01.005 28743363

[8] Van Houdenhove B Egle UT . Fibromyalgia: a stress disorder? Piecing the biopsychosocial puzzle together. Psychother Psychosom. (2004) 73:267–75. doi: 10.1159/000078843 15292624

[9] Diatchenko L Fillingim RB Smith SB Maixner W . The phenotypic and genetic signatures of common musculoskeletal pain conditions. Nat Rev Rheumatol. (2013) 9:340–50. doi: 10.1038/nrrheum.2013.43 23545734 PMC3991785

[10] Kwiatek R . Treatment of fibromyalgia. Aust Prescr. (2017) 40:179. doi: 10.18773/austprescr.2017.056 29109601 PMC5662432

[11] Goldenberg DL Clauw DJ Palmer RE Clair AG . Opioid use in fibromyalgia a cautionary tale. Mayo Clin Proc. (2016) 91:640–8. doi: 10.1016/j.mayocp.2016.02.002 26975749

[12] Alorfi NM . Pharmacological treatments of fibromyalgia in adults; overview of phase IV clinical trials. Front Pharmacol. (2022) 13:1017129. doi: 10.3389/fphar.2022.1017129 36210856 PMC9537626

[13] Seo S Jung YH Lee D Lee WJ Jang JH Lee JY . Abnormal neuroinflammation in fibromyalgia and CRPS using [11C]-(R)-PK11195 PET. PloS One. (2021) 16(2):e0246152. doi: 10.1371/journal.pone.0246152 33556139 PMC7870009

[14] Mueller C Fang YHD Jones C McConathy JE Raman F Lapi SE . Evidence of neuroinflammation in fibromyalgia syndrome: a [18F]DPA-714 positron emission tomography study. Pain. (2023) 164:2285–95. doi: 10.1097/j.pain.0000000000002927 37326674 PMC10502894

[15] Jung C Ichesco E Ratai EM Gonzalez RG Burdo T Loggia ML . Magnetic resonance imaging of neuroinflammation in chronic pain: a role for astrogliosis? Pain. (2020) 161:1555–64. doi: 10.1097/j.pain.0000000000001815 31990749 PMC7305954

[16] Quintero L Cuesta MC Silva JA Arcaya JL Pinerua-Suhaibar L Maixner W . Repeated swim stress increases pain-induced expression of c-Fos in the rat lumbar cord. Brain Res. (2003) 965:259–68. doi: 10.1016/s0006-8993(02)04224-5 12591144

[17] De La Luz-Cuellar YE Rodríguez-Palma EJ Franco-Enzástiga Ú Salinas-Abarca AB Delgado-Lezama R Granados-Soto V . Blockade of spinal α 5-GABA A receptors differentially reduces reserpine-induced fibromyalgia-type pain in female rats The role of spinal α 5 subunit-containing GABA A (α 5-GABA. (2019) 858:172443. doi: 10.1016/j.ejphar.2019.172443 31181208

[18] Qu TT Deng JX Li RL Cui ZJ Wang XQ Wang L . Stress injuries and autophagy in mouse hippocampus after chronic cold exposure. Neural Regener Res. (2017) 12:440–6. doi: 10.4103/1673-5374.202932 28469659 PMC5399722

[19] Lyons A Lynch AM Downer EJ Hanley R O’Sullivan JB Smith A . Fractalkine-induced activation of the phosphatidylinositol-3 kinase pathway attentuates microglial activation *in vivo* and *in vitro*. J Neurochem. (2009) 110:1547–56. doi: 10.1111/j.1471-4159.2009.06253.x 19627440

[20] Bajetto A Bonavia R Barbero S Schettini G . Characterization of chemokines and their receptors in the central nervous system: physiopathological implications. J Neurochem. (2002) 82:1311–29. doi: 10.1046/j.1471-4159.2002.01091.x 12354279

[21] Lee M Lee Y Song J Lee J Chang SY . Tissue-specific role of CX3CR1 expressing immune cells and their relationships with human disease. Immune Netw. (2018) 18:e5. doi: 10.4110/in.2018.18.e5 29503738 PMC5833124

[22] Clark AK Yip PK Malcangio M . The liberation of fractalkine in the dorsal horn requires microglial cathepsin S. J Neurosci. (2009) 29:6945–54. doi: 10.1523/jneurosci.0828-09.2009 19474321 PMC2698289

[23] Clark AK Staniland AA Malcangio M . Fractalkine/CX3CR1 signalling in chronic pain and inflammation. Curr Pharm Bio/Technol. (2011) 12:1707–14. doi: 10.2174/138920111798357465 21466443

[24] Pawelec P Ziemka-Nalecz M Sypecka J Zalewska T . The impact of the CX3CL1/CX3CR1 axis in neurological disorders. Cells. (2020) 9:2277. doi: 10.3390/cells9102277 33065974 PMC7600611

[25] Alarcón-Sánchez MA Becerra-Ruiz JS Guerrero-Velázquez C Mosaddad SA Heboyan A . The role of the CX3CL1/CX3CR1 axis as potential inflammatory biomarkers in subjects with periodontitis and rheumatoid arthritis: a systematic review. Immunity Inflammation Dis. (2024) 12:e1181. doi: 10.1002/iid3.1181 PMC10845211 38415821

[26] Jung S Aliberti J Graemmel P Sunshine MJ Kreutzberg GW Sher A . Analysis of fractalkine receptor CX3CR1 function by targeted deletion and green fluorescent protein reporter gene insertion. Mol Cell Biol. (2000) 20:4106–14. doi: 10.1128/mcb.20.11.4106-4114.2000 10805752 PMC85780

[27] Fülöp B Hunyady Á Bencze N Kormos V Szentes N Dénes Á . IL-1 mediates chronic stress-induced hyperalgesia accompanied by microglia and astroglia morphological changes in pain-related brain regions in mice. Int J Mol Sci. (2023) 24:5479. doi: 10.3390/ijms24065479 36982563 PMC10052634

[28] Ihne JL Fitzgerald PJ Hefner KR Holmes A . Pharmacological modulation of stress-induced behavioral changes in the light/dark exploration test in male C57BL/6J mice. Neuropharmacology. (2012) 62(1):464–73. doi: 10.1016/j.neuropharm.2011.08.045 21906605 PMC3195838

[29] Wollberg AR Ericsson-Dahlstrand A Juréus A Ekerot P Simon S Nilsson M . Pharmacological inhibition of the chemokine receptor CX3CR1 attenuates disease in a chronic-relapsing rat model for multiple sclerosis. Proc Natl Acad Sci USA. (2014) 111:5409–14. doi: 10.1073/pnas.1316510111 24706865 PMC3986185

[30] Borbély É Botz B Bölcskei K Kenyér T Kereskai L Kiss T . Capsaicin-sensitive sensory nerves exert complex regulatory functions in the serum-transfer mouse model of autoimmune arthritis. Brain Behav Immun. (2015) 45:50–9. doi: 10.1016/j.bbi.2014.12.012 PMC4349500 25524130

[31] Tékus V Hajna Z Borbély É Markovics A Bagoly T Szolcsányi J . A CRPS-IgG-transfer-trauma model reproducing inflammatory and positive sensory signs associated with complex regional pain syndrome. Pain. (2014) 155:299–308. doi: 10.1016/j.pain.2013.10.011 24145209

[32] Gould TD Dao DT Kovacsics CE . The open field test. In: Neuromethods. Humana Press (2009). p. 1–20.

[33] Steru L Chermat R Thierry B Simon P . The tail suspension test: a new method for screening antidepressants in mice. Psychopharmacol (Berl). (1985) 85:367–70. doi: 10.1007/bf00428203 3923523

[34] Ghasemi M Montaser-Kouhsari L Shafaroodi H Nezami BG Ebrahimi F Dehpour AR . NMDA receptor/nitrergic system blockage augments antidepressant-like effects of paroxetine in the mouse forced swimming test. Psychopharmacol (Berl). (2009) 206:325–33. doi: 10.1007/s00213-009-1609-1 19609507

[35] Dombi Á Sánta C Bátai IZ Kormos V Kecskés A Tékus V . Dimethyl trisulfide diminishes traumatic neuropathic pain acting on TRPA1 receptors in mice. Int J Mol Sci. (2021) 22:3363. doi: 10.3390/ijms22073363 33806000 PMC8036544

[36] Cserép C Pósfai B Lénárt N Fekete R László ZI Lele Z . Microglia monitor and protect neuronal function through specialized somatic purinergic junctions. Science (80-). (2020) 367(6477):528–37. doi: 10.2139/ssrn.3339900 31831638

[37] Schindelin J Arganda-Carreras I Frise E Kaynig V Longair M Pietzsch T . Fiji: an open-source platform for biological-image analysis. Nat Methods. (2012) 9:676–82. doi: 10.1038/nmeth.2019 22743772 PMC3855844

[38] Harrison L Pfuhlmann K Schriever SC Pfluger PT . Profound weight loss induces reactive astrogliosis in the arcuate nucleus of obese mice. Mol Metab. (2019) 24:149–55. doi: 10.1016/j.molmet.2019.03.009 30979678 PMC6977167

[39] Barrett AC Smith ES Picker MJ . Sex-related differences in mechanical nociception and antinociception produced by μ- and κ-opioid receptor agonists in rats. Eur J Pharmacol. (2002) 452:163–73. doi: 10.1016/s0014-2999(02)02274-4 12354566

[40] Bourgeois JR Feustel PJ Kopec AM . Sex differences in choice-based thermal nociceptive tests in adult rats. Behav Brain Res. (2022) 429:113919. doi: 10.1016/j.bbr.2022.113919 35525338

[41] Body weight information for C57BL/6J | The jackson laboratory. doi: 10.1007/3-540-29623-9_6364

[42] Han J Fan Y Zhou K Blomgren K Harris RA . Uncovering sex differences of rodent microglia. J Neuroinflamm. (2021) 18:1–11. doi: 10.1186/s12974-021-02124-z 33731174 PMC7972194

[43] Lynch MA . Exploring sex-related differences in microglia may be a game-changer in precision medicine. Front Aging Neurosci. (2022) 14:868448. doi: 10.3389/fnagi.2022.868448 35431903 PMC9009390

[44] Gozlan E Lewit-Cohen Y Frenkel D . Sex differences in astrocyte activity. Cells. (2024) 13:1724. doi: 10.3390/cells13201724 39451242 PMC11506538

[45] Zhang AY Elias E Manners MT . Sex-dependent astrocyte reactivity: unveiling chronic stress-induced morphological changes across multiple brain regions. Neurobiol Dis. (2024) 200:106610. doi: 10.1016/j.nbd.2024.106610 39032799 PMC11500746

[46] Gupta A Silman AJ . Psychological stress and fibromyalgia: a review of the evidence suggesting a neuroendocrine link. Arthritis Res Ther. (2004) 6:98. doi: 10.1186/ar1176 15142258 PMC416451

[47] Barke A . Chronic pain has arrived in the ICD-11. In: Isap - Int Assoc Study Pain (2019).

[48] Wolfe F Walitt B Perrot S Rasker JJ Häuser W . Fibromyalgia diagnosis and biased assessment: Sex, prevalence and bias. PloS One. (2018) 13:e0203755. doi: 10.1371/journal.pone.0203755 30212526 PMC6136749

[49] Paulson M Danielson E Larsson K Norberg A . Men’s descriptions of their experience of nonmalignant pain of fibromyalgia type. Scand J Caring Sci. (2001) 15:54–9. doi: 10.1046/j.1471-6712.2001.1510054.x 37945311

[50] Alter BJ Moses M DeSensi R O’Connell B Bernstein C McDermott S . Hierarchical clustering applied to chronic pain drawings identifies undiagnosed fibromyalgia: Implications for busy clinical practice. J Pain. (2024) 25:104489. doi: 10.1016/j.jpain.2024.02.003 38354967 PMC11180596

[51] Goshen I Kreisel T Ben-Menachem-Zidon O Licht T Weidenfeld J Ben-Hur T . Brain interleukin-1 mediates chronic stress-induced depression in mice via adrenocortical activation and hippocampal neurogenesis suppression. Mol Psychiatry. (2008) 13:717–28. doi: 10.1038/sj.mp.4002055 17700577

[52] Tynan RJ Naicker S Hinwood M Nalivaiko E Buller KM Pow DV . Chronic stress alters the density and morphology of microglia in a subset of stress-responsive brain regions. Brain Behav Immun. (2010) 24:1058–68. doi: 10.1016/j.bbi.2010.02.001 20153418

[53] Blandino P Barnum CJ Deak T . The involvement of norepinephrine and microglia in hypothalamic and splenic IL-1beta responses to stress. J Neuroimmunol. (2006) 173:87–95. doi: 10.1016/j.jneuroim.2005.11.021 16386803

[54] Fülöp B Borbély É Helyes Z . How does chronic psychosocial distress induce pain? Focus on neuroinflammation and neuroplasticity changes. Brain Behav Immun - Heal. (2025) 44:100964. doi: 10.1016/j.bbih.2025.100964 PMC11875130 40034488

[55] Nagakura Y Miwa M Yoshida M Miura R Tanei S Tsuji M . Spontaneous pain-associated facial expression and efficacy of clinically used drugs in the reserpine-induced rat model of fibromyalgia. Eur J Pharmacol. (2019) 864:172716. doi: 10.1016/j.ejphar.2019.172716 31589868

[56] Nagakura Y Oe T Aoki T Matsuoka N . Biogenic amine depletion causes chronic muscular pain and tactile allodynia accompanied by depression: A putative animal model of fibromyalgia. Pain. (2009) 146:26–33. doi: 10.1016/j.pain.2009.05.024 19646816

[57] Sluka KA Kalra A Moore SA . Unilateral intramuscular injections of acidic saline produce a bilateral, long-lasting hyperalgesia. Muscle Nerve. (2001) 24(1):37–46. doi: 10.1002/1097-4598(200101)24:1<37::aid-mus4>3.0.co;2-8 11150964

[58] Fernández-Arjona MM Grondona JM Fernández-Llebrez P López-Ávalos MD . Microglial morphometric parameters correlate with the expression level of IL-1β, and allow identifying different activated morphotypes. Front Cell Neurosci. (2019) 13:472. doi: 10.3389/fncel.2019.00472 31708746 PMC6824358

[59] Bollinger JL Bergeon Burns CM Wellman CL . Differential effects of stress on microglial cell activation in male and female medial prefrontal cortex. Brain Behav Immun. (2016) 52:88–97. doi: 10.1016/j.bbi.2015.10.003 26441134 PMC4909118

[60] Bollinger JL Collins KE Patel R Wellman CL . Behavioral stress alters corticolimbic microglia in a sex- and brain region-specific manner. PloS One. (2017) 12(12):e0187631. doi: 10.1371/journal.pone.0187631 29194444 PMC5711022

[61] Patani R Hardingham GE Liddelow SA . Functional roles of reactive astrocytes in neuroinflammation and neurodegeneration. Nat Rev Neurol. (2023) 19:395–409. doi: 10.1038/s41582-023-00822-1 37308616

[62] Liddelow SA Guttenplan KA Clarke LE Bennett FC Bohlen CJ Schirmer L . Neurotoxic reactive astrocytes are induced by activated microglia. Nature. (2017) 541:481–7. doi: 10.1038/nature21029 28099414 PMC5404890

[63] Molina VA Heyser CJ Spear LP . Chronic variable stress or chronic morphine facilitates immobility in a forced swim test: Reversal by naloxone. Psychopharmacol (Berl). (1994) 114:433–40. doi: 10.1007/bf02249333 7855201

[64] Suvrathan A Tomar A Chattarji S . Effects of chronic and acute stress on rat behaviour in the forced-swim test. Stress. (2010) 13:533–40. doi: 10.3109/10253890.2010.489978 20666651

[65] Sequeira-Cordero A Salas-Bastos A Fornaguera J Brenes JC . Behavioural characterisation of chronic unpredictable stress based on ethologically relevant paradigms in rats. Sci Rep. (2019) 9:1–21. doi: 10.1038/s41598-019-53624-1 31758000 PMC6874551

[66] Ge MJ Chen G Zhang ZQ Yu ZH Shen JX Pan C . Chronic restraint stress induces depression-like behaviors and alterations in the afferent projections of medial prefrontal cortex from multiple brain regions in mice. Brain Res Bull. (2024) 213:110981. doi: 10.1016/j.brainresbull.2024.110981 38777132

[67] McCully ML Kouzeli A Moser B . Peripheral tissue chemokines: Homeostatic control of immune surveillance T cells. Trends Immunol. (2018) 39:734–47. doi: 10.1016/j.it.2018.06.003 30001872

[68] Zhan L Qiu M Zheng J Lai M Lin K Dai J . Fractalkine/CX3CR1 axis is critical for neuroprotection induced by hypoxic postconditioning against cerebral ischemic injury. Cell Commun Signal. (2024) 22:1–18. doi: 10.1186/s12964-024-01830-4 39327578 PMC11426015

[69] Paul D Basavan D . Implications of fractalkine on glial function, ablation and glial proteins/receptors/markers—understanding its therapeutic usefulness in neurological settings: a narrative review. Futur J Pharm Sci. (2022) 8:1–29. doi: 10.1186/s43094-022-00446-0 38164791

[70] Corsi G Picard K di Castro MA Garofalo S Tucci F Chece G . Microglia modulate hippocampal synaptic transmission and sleep duration along the light/dark cycle. Glia. (2022) 70:89–105. doi: 10.1002/glia.24090 34487590 PMC9291950

[71] Zhan Y Paolicelli RC Sforazzini F Weinhard L Bolasco G Pagani F . Deficient neuron-microglia signaling results in impaired functional brain connectivity and social behavior. Nat Neurosci. (2014) 17:400–6. doi: 10.1038/nn.3641 24487234

[72] You H Baluszek S Kaminska B . Supportive roles of brain macrophages in CNS metastases and assessment of new approaches targeting their functions. Theranostics. (2020) 10:2949. doi: 10.7150/thno.40783 32194848 PMC7053204

[73] Rua R McGavern DB . Advances in meningeal immunity. Trends Mol Med. (2018) 24:542–59. doi: 10.1016/j.molmed.2018.04.003 29731353 PMC6044730

[74] Gyoneva S Hosur R Gosselin D Zhang B Ouyang Z Cotleur AC . Cx3cr1-deficient microglia exhibit a premature aging transcriptome. Life Sci Alliance. (2019) 2:e201900453. doi: 10.26508/lsa.201900453 31792059 PMC6892408

[75] Liang KJ Lee JE Wang YD Ma W Fontainhas AM Fariss RN . Regulation of dynamic behavior of retinal microglia by CX3CR1 signaling. Invest Ophthalmol Vis Sci. (2009) 50:4444–51. doi: 10.1167/iovs.08-3357 19443728 PMC2749316

[76] Hoshiko M Arnoux I Avignone E Yamamoto N Audinat E . Deficiency of the microglial receptor CX3CR1 impairs postnatal functional development of thalamocortical synapses in the barrel cortex. J Neurosci. (2012) 32:15106–11. doi: 10.1523/jneurosci.1167-12.2012 23100431 PMC6704837

[77] Pagani F Paolicelli RC Murana E Cortese B Di Angelantonio S Zurolo E . Defective microglial development in the hippocampus of Cx3cr1 deficient mice. Front Cell Neurosci. (2015) 9:133241. doi: 10.3389/fncel.2015.00111 25873863 PMC4379915

[78] Cho SH Sun B Zhou Y Kauppinen TM Halabisky B Wes P . CX3CR1 protein signaling modulates microglial activation and protects against plaque-independent cognitive deficits in a mouse model of Alzheimer disease. J Biol Chem. (2011) 286:32713–22. doi: 10.1074/jbc.m111.254268 21771791 PMC3173153

[79] Lee S Varvel NH Konerth ME Xu G Cardona AE Ransohoff RM . CX3CR1 deficiency alters microglial activation and reduces beta-amyloid deposition in two Alzheimer’s disease mouse models. Am J Pathol. (2010) 177:2549. doi: 10.2353/ajpath.2010.100265 20864679 PMC2966811

[80] Bridge S Karagiannis SN Borsini A . The complex role of the chemokine CX3CL1/Fractalkine in major depressive disorder: A narrative review of preclinical and clinical studies. Brain Behav Immun - Heal. (2024) 38:100778. doi: 10.1016/j.bbih.2024.100778 38706575 PMC11070239

[81] Wohleb ES Powell ND Godbout JP Sheridan JF . Stress-induced recruitment of bone marrow-derived monocytes to the brain promotes anxiety-like behavior. J Neurosci. (2013) 33:13820–33. doi: 10.1523/jneurosci.1671-13.2013 23966702 PMC3755721

[82] Liu Y Zhang T Meng D Sun L Yang G He Y . Involvement of CX3CL1/CX3CR1 in depression and cognitive impairment induced by chronic unpredictable stress and relevant underlying mechanism. Behav Brain Res. (2020) 381:112371. doi: 10.1016/j.bbr.2019.112371 31765724

[83] Hellwig S Brioschi S Dieni S Frings L Masuch A Blank T . Altered microglia morphology and higher resilience to stress-induced depression-like behavior in CX3CR1-deficient mice. Brain Behav Immun. (2016) 55:126–37. doi: 10.1016/j.bbi.2015.11.008 26576722

[84] Rogers JT Morganti JM Bachstetter AD Hudson CE Peters MM Grimmig BA . CX3CR1 deficiency leads to impairment of hippocampal cognitive function and synaptic plasticity. J Neurosci. (2011) 31:16241–50. doi: 10.1523/jneurosci.3667-11.2011 22072675 PMC3236509

[85] Winkler Z Kuti D Ferenczi S Gulyás K Polyák Á Kovács KJ . Impaired microglia fractalkine signaling affects stress reaction and coping style in mice. Behav Brain Res. (2017) 334:119–28. doi: 10.1016/j.bbr.2017.07.023 28736330

[86] Bäckryd E Tanum L Lind AL Larsson A Gordh T . Evidence of both systemic inflammation and neuroinflammation in fibromyalgia patients, as assessed by a multiplex protein panel applied to the cerebrospinal fluid and to plasma. J Pain Res. (2017) 10:515–25. doi: 10.2147/JPR.S128508 PMC5344444 28424559

[87] Silva R Malcangio M . Fractalkine/CX3CR1 pathway in neuropathic pain: An update. Front Pain Res. (2021) 2:684684. doi: 10.3389/fpain.2021.684684 35295489 PMC8915718

[88] Gu N Yi MH Murugan M Xie M Parusel S Peng J . Spinal microglia contribute to sustained inflammatory pain via amplifying neuronal activity. Mol Brain. (2022) 15(1):86. doi: 10.1186/s13041-022-00970-3 36289499 PMC9609165

[89] Staniland AA Clark AK Wodarski R Sasso O Maione F D’Acquisto F . Reduced inflammatory and neuropathic pain and decreased spinal microglial response in fractalkine receptor (CX3CR1) knockout mice. J Neurochem. (2010) 114:1143–57. doi: 10.1111/j.1471-4159.2010.06837.x 20524966

[90] Milligan E Zapata V Schoeniger D Chacur M Green P Poole S . An initial investigation of spinal mechanisms underlying pain enhancement induced by fractalkine, a neuronally released chemokine. Eur J Neurosci. (2005) 22:2775–82. doi: 10.1111/j.1460-9568.2005.04470.x 16324111

[91] Sessler K Blechschmidt V Hoheisel U Mense S Schirmer L Treede RD . Spinal cord fractalkine (CX3CL1) signaling is critical for neuronal sensitization in experimental nonspecific, myofascial low back pain. J Neurophysiol. (2021) 125:1598–611. doi: 10.1152/jn.00348.2020 33596743

[92] Hinwood M Kluge MG Ilicic M Walker FR . Understanding microglial involvement in stress-induced mood disturbance: a modulator of vulnerability? Curr Opin Behav Sci. (2019) 28:98–104. doi: 10.1016/j.cobeha.2019.01.001 38826717

[93] Rimmerman N Schottlender N Reshef R Dan-Goor N Yirmiya R . The hippocampal transcriptomic signature of stress resilience in mice with microglial fractalkine receptor (CX3CR1) deficiency. Brain Behav Immun. (2017) 61:184–96. doi: 10.1016/j.bbi.2016.11.023 27890560

[94] Cserép C Schwarcz AD Pósfai B László ZI Kellermayer A Környei Z . Microglial control of neuronal development via somatic purinergic junctions. Cell Rep. (2022) 40:111369. doi: 10.1016/j.celrep.2022.111369 36130488 PMC9513806

[95] Cserép C Pósfai B Dénes Á . Shaping neuronal fate: Functional heterogeneity of direct microglia-neuron interactions. Neuron. (2021) 109:222–40. doi: 10.1016/j.neuron.2020.11.007 33271068

[96] Pósfai B Szabadits E Cserép C Vida S Schwarcz AD Fekete R . P2Y12 receptor function governs microglial surveillance and cell-cell interactions in the cerebral cortex. Glia. (2026) 74(2):e70109. 41331281 10.1002/glia.70109

[97] Paolicelli RC Bolasco G Pagani F Maggi L Scianni M Panzanelli P . Synaptic pruning by microglia is necessary for normal brain development. Science. (2011) 333:1456–8. doi: 10.1126/science.1202529 21778362

[98] Szepesi Z Manouchehrian O Bachiller S Deierborg T . Bidirectional microglia–neuron communication in health and disease. Front Cell Neurosci. (2018) 12:323. doi: 10.3389/fncel.2018.00323 30319362 PMC6170615

[99] Eyo UB Wu LJ . Bidirectional microglia-neuron communication in the healthy brain. Neural Plast. (2013) 2013:456857. doi: 10.1155/2013/456857 24078884 PMC3775394

[100] York EM Bernier LP MacVicar BA . Microglial modulation of neuronal activity in the healthy brain. Dev Neurobiol. (2018) 78:593–603. doi: 10.1002/dneu.22571 29271125

[101] Marciante AB Tadjalli A Nikodemova M Burrowes KA Oberto J Luca EK . Microglia regulate motor neuron plasticity via reciprocal fractalkine and adenosine signaling. Nat Commun. (2024) 15:1–16. doi: 10.1038/s41467-024-54619-x 39609435 PMC11605081

[102] Ueno M Fujita Y Tanaka T Nakamura Y Kikuta J Ishii M . Layer V cortical neurons require microglial support for survival during postnatal development. Nat Neurosci. (2013) 16:543–51. doi: 10.1038/nn.3358 23525041

[103] Röhl C Lucius R Sievers J . The effect of activated microglia on astrogliosis parameters in astrocyte cultures. Brain Res. (2007) 1129:43–52. doi: 10.1016/j.brainres.2006.10.057 17169340

[104] Study details | A study to evaluate the safety of KAND567, in combination with carboplatin therapy, in women with recurrent epithelial ovarian, fallopian tube, or primary peritoneal cancer | ClinicalTrials.gov. Available online at: https://clinicaltrials.gov/study/NCT06087289.

[105] Meyer RA Ringkamp M Campbell JN Raja SN . Peripheral mechanisms of cutaneous nociception. In: Wall Melzack’s Textb Pain (2006). p. 3–34.

[106] Karlström S Nordvall G Sohn D Hettman A Turek D Åhlin K . Substituted 7-amino-5-thio-thiazolo[4,5-d]pyrimidines as potent and selective antagonists of the fractalkine receptor (CX3CR1). J Med Chem. (2013) 56:3177–90. doi: 10.1021/jm3012273 23516963

[107] Dudek KA Dion-Albert L Lebel M LeClair K Labrecque S Tuck E . Molecular adaptations of the blood–brain barrier promote stress resilience vs. depression. Proc Natl Acad Sci USA. (2020) 117:3326–36. doi: 10.1073/pnas.1914655117 31974313 PMC7022213

[108] Lehmann ML Weigel TK Cooper HA Elkahloun AG Kigar SL Herkenham M . Decoding microglia responses to psychosocial stress reveals blood-brain barrier breakdown that may drive stress susceptibility. Sci Rep. (2018) 8(1):11240. doi: 10.1038/s41598-018-28737-8 30050134 PMC6062609

[109] Ashmawi HA Freire GMG . Peripheral and central sensitization. Rev Dor. (2016) 17:31–4. doi: 10.5935/1806-0013.20160044

[110] Baron R Hans G Dickenson AH . Peripheral input and its importance for central sensitization. Ann Neurol. (2013) 74:630–6. doi: 10.1002/ana.24017 24018757

[111] Nemska S Gassmann M Bang ML Frossard N Tavakoli R . Antagonizing the CX3CR1 receptor markedly reduces development of cardiac hypertrophy after transverse aortic constriction in mice. J Cardiovasc Pharmacol. (2021) 78:792–801. doi: 10.1097/fjc.0000000000001130 34882111

[112] Zhou Y Zhang L Hao Y Yang L Fan S Xiao Z . FKN/CX3CR1 axis facilitates migraine-like behaviour by activating thalamic-cortical network microglia in status epilepticus model rats. J Headache Pain. (2022) 23:1–16. doi: 10.1186/s10194-022-01416-w 35382731 PMC8981829

[113] Inan S Chen X Eisenstein EM Meissler JJ Geller EB Tallarida C . Chemokine receptor antagonists enhance morphine’s antinociceptive effect but not respiratory depression. Life Sci. (2021) 285:120014. doi: 10.1016/j.lfs.2021.120014 34619167

[114] El-Brolosy MA Stainier DYR . Genetic compensation: A phenomenon in search of mechanisms. PloS Genet. (2017) 13:e1006780. doi: 10.1371/journal.pgen.1006780 28704371 PMC5509088

