# Supplementary material for: Microglial CX3CR1 signaling mediates stress-induced pain behavior in mice
Source: Front Immunol. 2026 Jul 1;17:1869876. doi: 10.3389/fimmu.2026.1869876 (PMC13368685; doi:10.3389/fimmu.2026.1869876)
Supplement: Supplementary file 1 [file DataSheet1.docx]

***Supplementary Material***


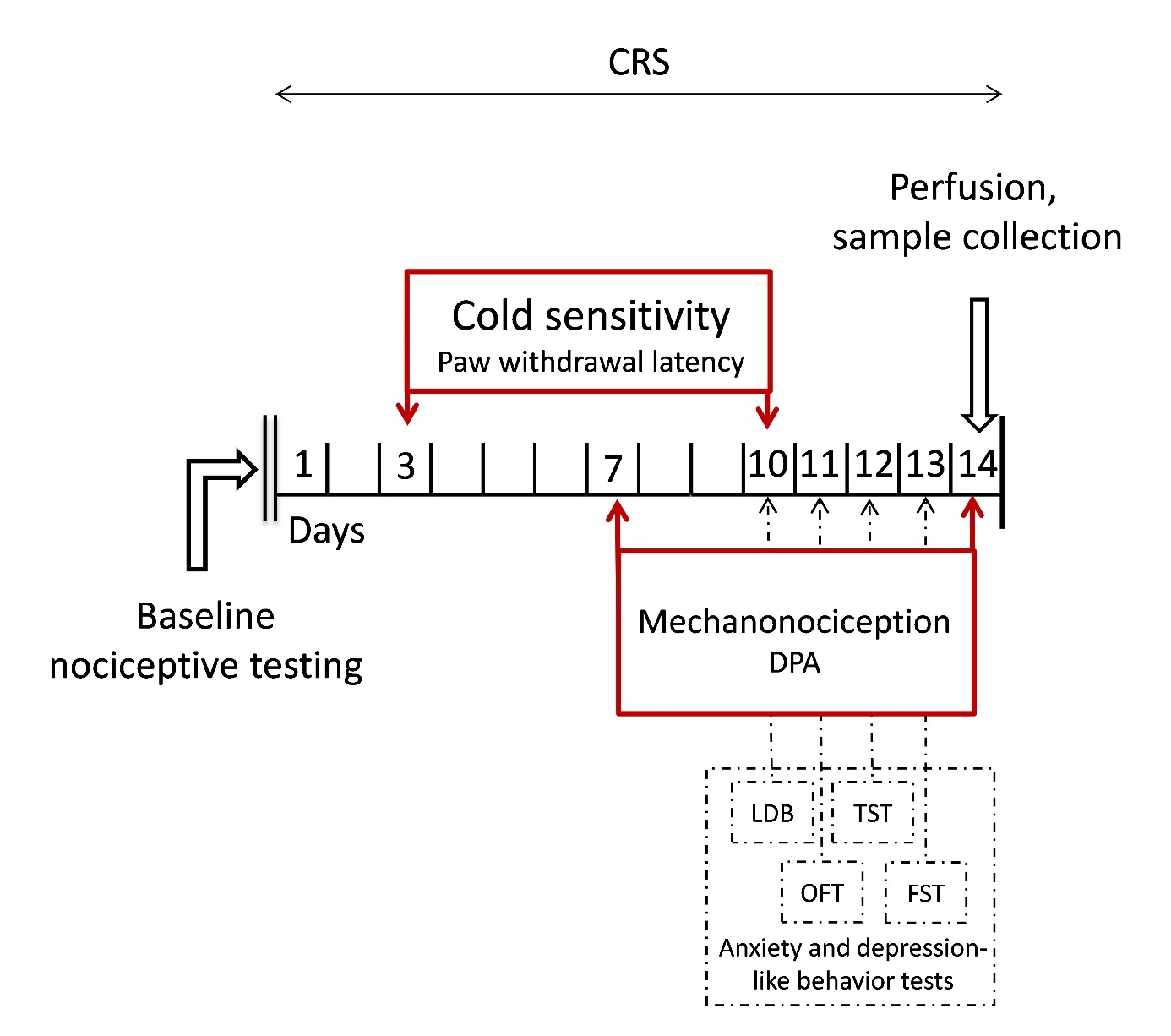


**Supplementary Figure 1.** Experimental design including the timing of the baseline measurements, chronic restraint stress (CRS), nociceptive and behavioral tests, and perfusion-tissue processing. DPA: dynamic plantar aesthesiometry; LDB: light–dark box test; OFT: open field test; TST: tail suspension test; FST: forced swim test.


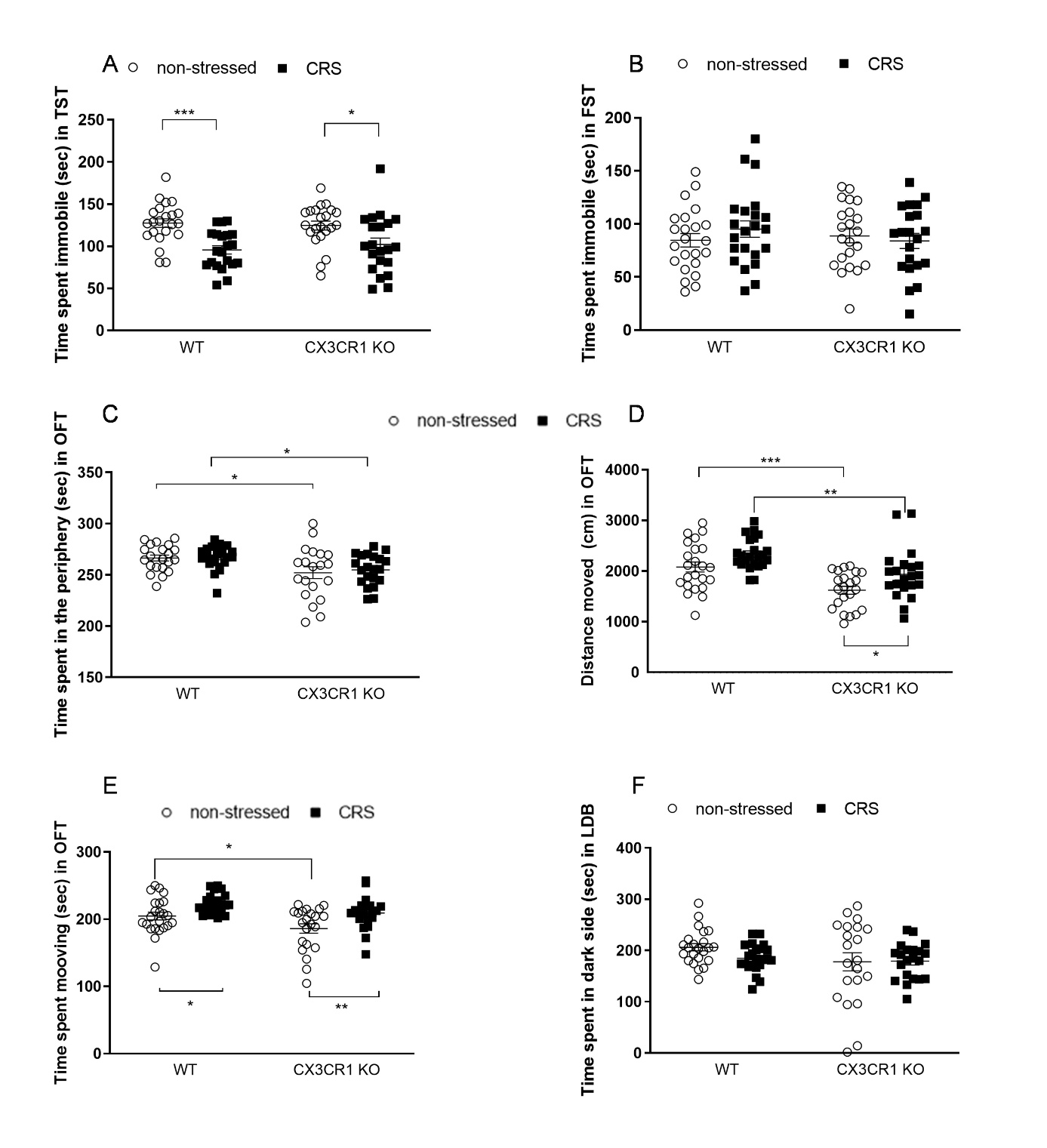


**Supplementary Figure 2.** Effects of chronic restraint stress (CRS) on anxiety- and depression-like behavior in wild-type (WT) and CX3CR1 knock-out (KO) merged male and female animals. Tail suspension test (TST, A) and forced swim test (FST, B) shows time spent immobile after 2 weeks of CRS. In the open field test (OFT), the time spent in center (C), the distance moved (D) and the time spent moving (E) is presented. In the light–dark box test (LDB, F), the time spent in the lit compartment is shown. Data are presented as the means ± SEM of n = 21–23 animals with individual plots; two-way analysis of variance (ANOVA), followed by Tukey’s tests; * p<0.05, **p<0.01, ***p<0.001 vs. indicated groups.


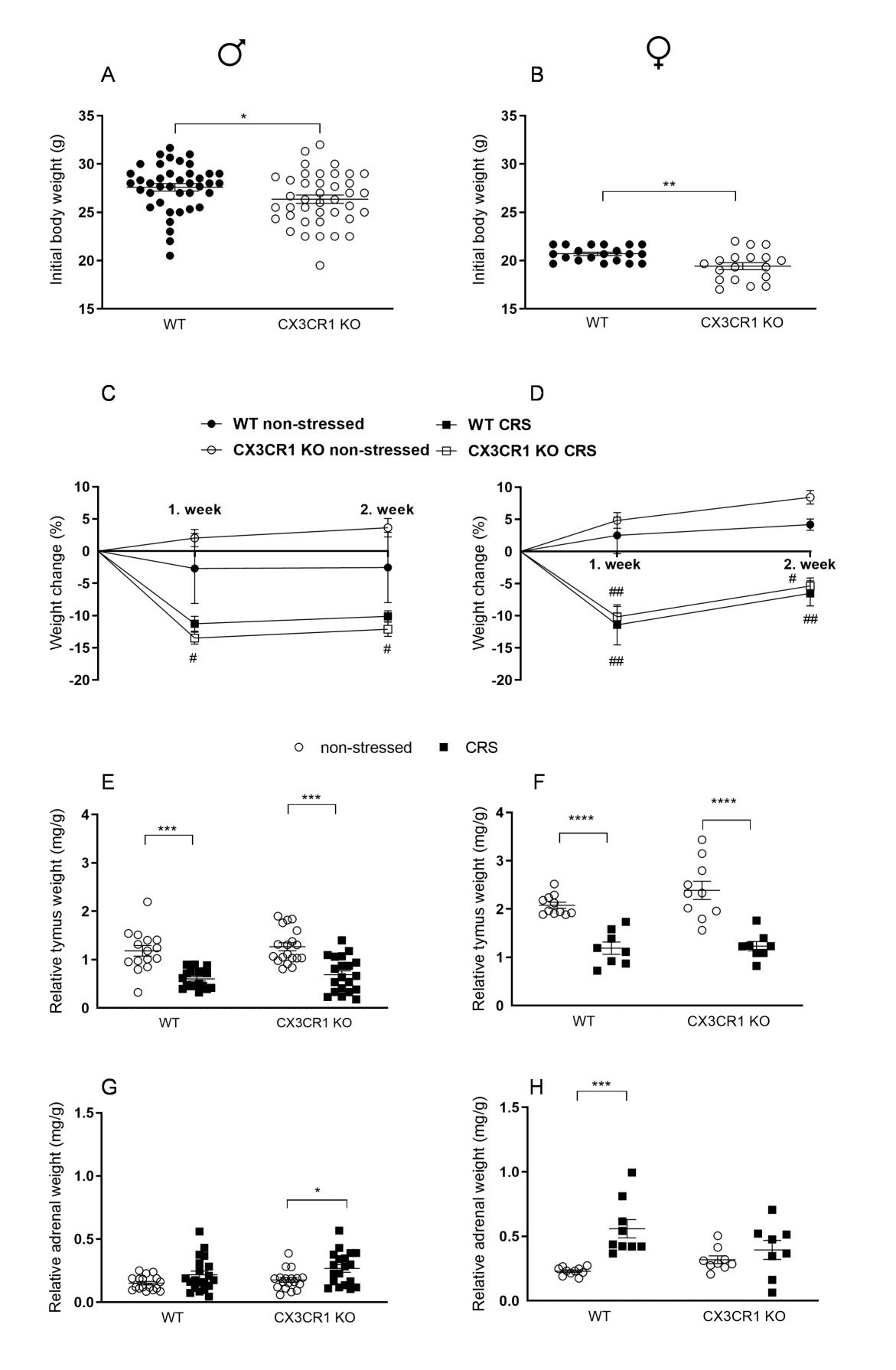


**Supplementary Figure 3.** Initial body weights of male and female wild-type (WT) and CX3CR1 knock-out (KO) mice (A, B). Data are presented as the mean ± SEM (n=23-40), animals with individual plots. Unpaired t-test, *p<0.05, **p<0.01 vs. indicated groups.
Effects of chronic restraint stress (CRS) on body weight of male (C), female (D) animals, Data are presented as the means ± SEM of n=4–19 animals; two-way repeated measurement analysis of variance (ANOVA), followed by Sidak’s tests; #p< 0.001, p##<0.0001 vs. respective non-stressed group.
Relative tymus and adrenal gland weights of male (E,G), female (F,H) animals with individual plots. Data are presented as the means ± SEM of n=4–19 animals; two-way analysis of variance (ANOVA), followed by Tukey’s tests*p<0.05, **p<0.01, ***p<0.001, ****p<0.0001 vs. indicated groups.


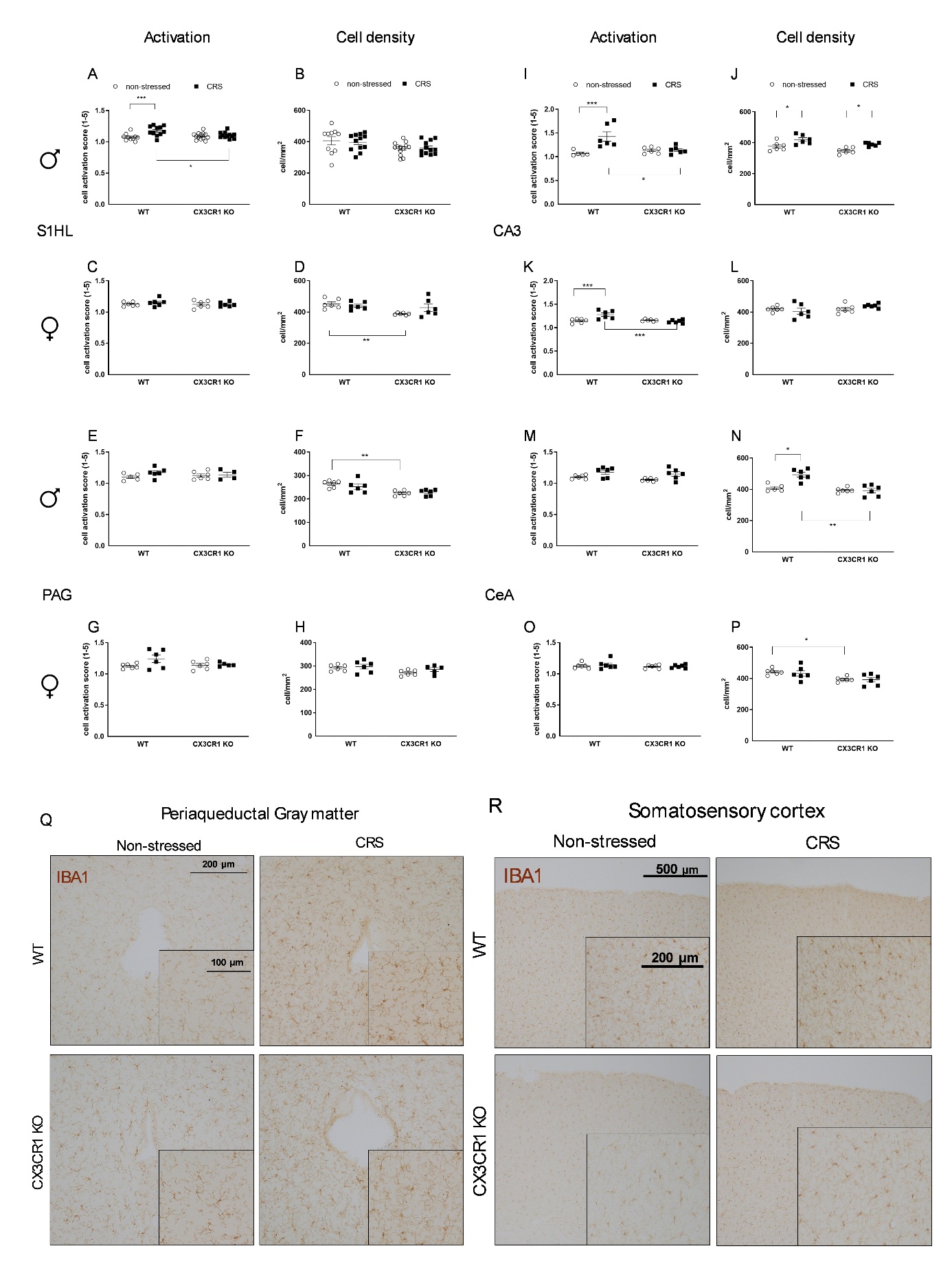


**Supplementary Figure 4.** Effects of chronic restraint stress (CRS) on ionized calcium binding adapter protein 1 (IBA1) positive cell activation and density in the somatosensory cortex (S1HL; A,B,C,D), periaqueductal gray matter (PAG; E,F,G,H), hippocampus Cornu Ammonis 3 (CA3; I,J,K,L) and, central amygdala (CeA; M,N,O,P) of male and female wild-type (WT) and CX3CR1 knock-out (KO) mice. Data are presented as the mean ± SEM, animals with individual plots (n=6/group). Two-way analysis of variance (ANOVA), followed by Tukey’s tests; *p< 0.05, **p<0.01, ***p<0.001 vs. indicated groups. Representative images show the IBA1+ microglia cells in Periaqueductal Gray matter (Q) and Somatosensory cortex in female animals’ samples (R)


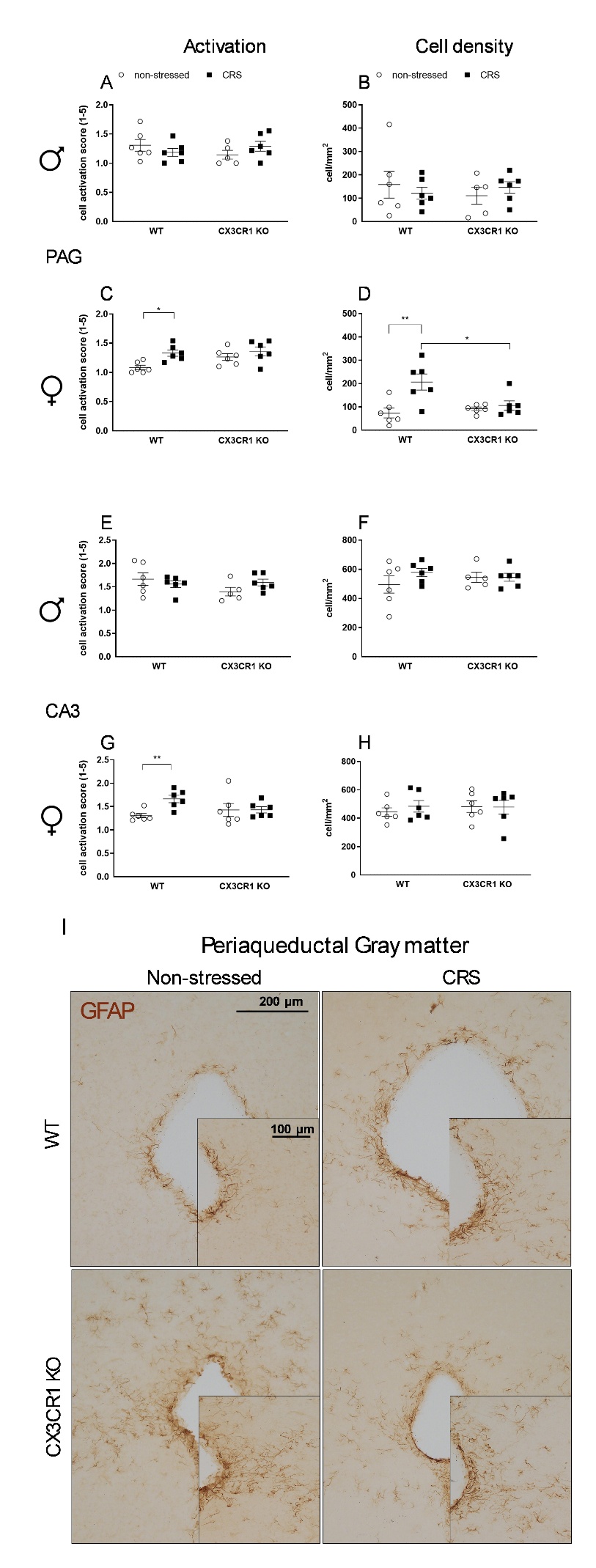


**Supplementary Figure 5.** Effects of chronic restraint stress (CRS) on glial fibrillary acidic protein (GFAP) positive cell activation and density in the periaqueductal gray matter (PAG; A, B, C, D), hippocampus Cornu Ammonis 3 (CA3; E, F, G, H) of male and female wild-type (WT) and CX3CR1 knock-out (KO) mice. Data are presented as the mean ± SEM, animals with individual plots (n=6/group). Two-way analysis of variance (ANOVA), followed by Tukey’s tests; *p<0.05, **p<0.01 vs. indicated groups. Representative images show the GFAP+ astrocyte cells in Periaqueductal Gray matter in female mice (I)


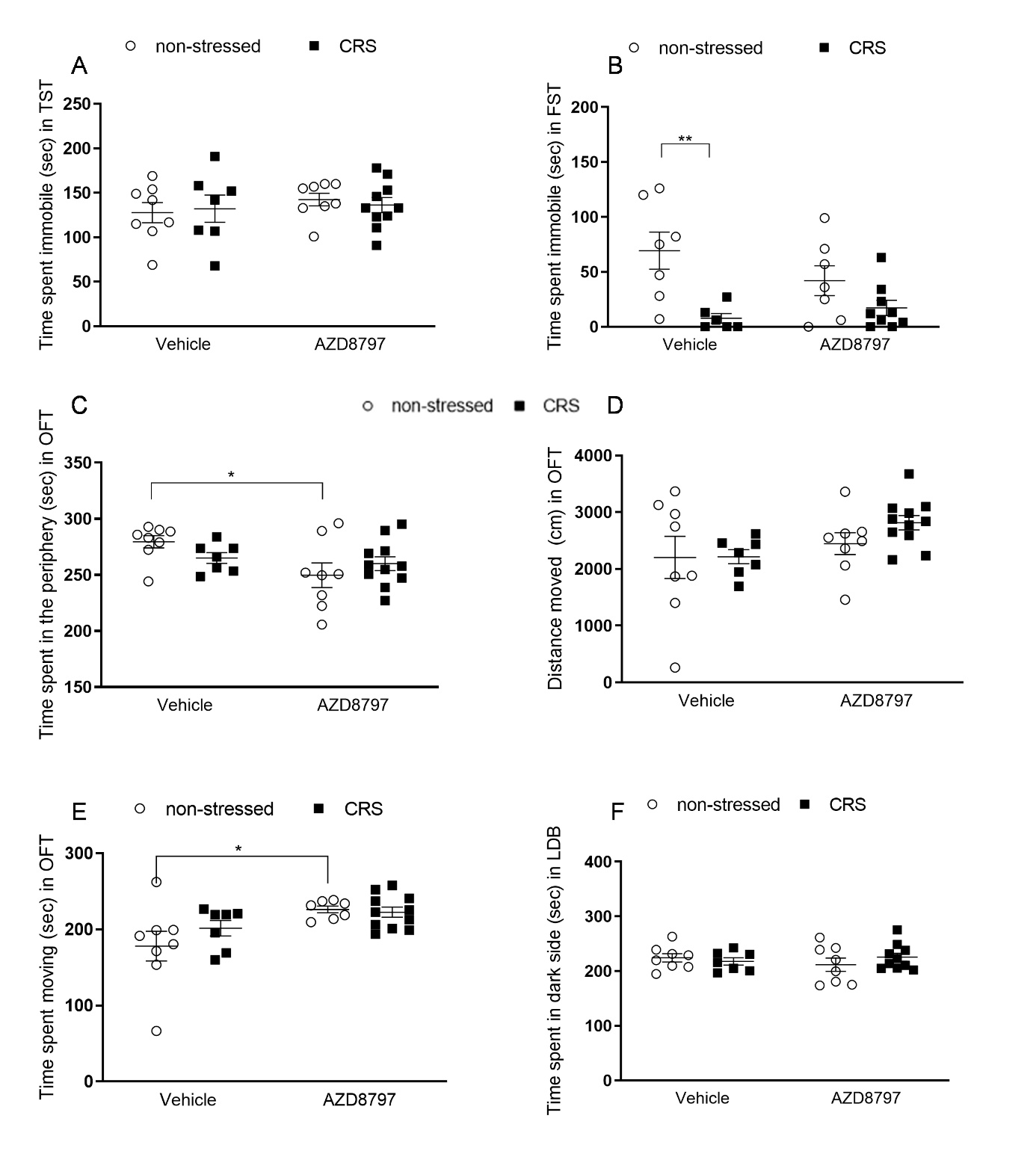


**Supplementary Figure 6.** Effects of chronic restraint stress (CRS) on anxiety- and depression-like behavior in Vehicle and AZD8797-treated male mice. Tail suspension test (TST, A) and forced swim test (FST, B) shows time spent immobile after 2 weeks of CRS. In th open field test (OFT), the time spent in center (C), the distance moved (D) and the time spent moving (E) is presented. In the light–dark box test (LDB, F), the time spent in the lit compartment is shown. Data are presented as the means ± SEM of n = 21–23 animals with individual plots; two-way analysis of variance (ANOVA), followed by Tukey’s tests; *p<0.05, **p<0.01, ***p<0.001 vs. indicated groups.


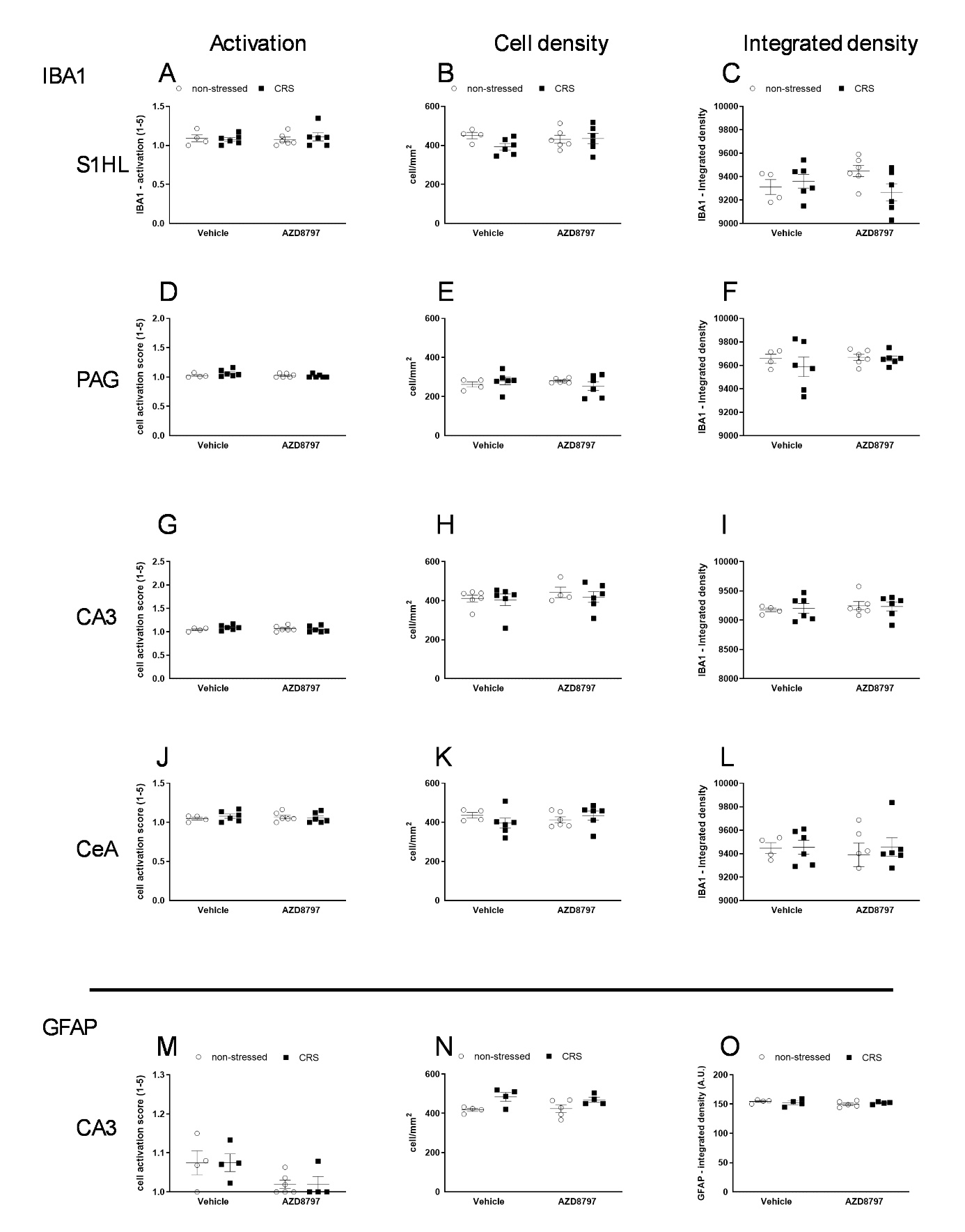


**Supplementary Figure 7.** Effects of chronic restraint stress (CRS) on ionized calcium binding adapter protein 1 (IBA1) positive cell activation, density and integrated density in the somatosensory cortex (S1HL; A, B, C), periaqueductal gray matter (PAG; D, E, F), hippocampus Cornu Ammonis 3 (CA3; G, H, I) and, central amygdala (CeA; J, K, L) of male vehicle and AZD8797-treated mice.
Glial fibrillary acidic protein (GFAP) positive cell activation, density and integrated density in the CA3 (M, N, O) after 2 weeks CRS protocol, of male vehicle and AZD8797-treated mice. Data are presented as the mean ± SEM, animals with individual plots (n=5-6/group). Two-way analysis of variance (ANOVA), followed by Tukey’s tests.

|  | **Male** | | | | **Female** | | | |
| --- | --- | --- | --- | --- | --- | --- | --- | --- |
|  | **WT non-stressed vs. WT CRS** | ***CX3CR1 KO* non-stressed vs. *CX3CR1 KO CRS*** | **WT non-stressed vs. *CX3CR1 KO* non-stressed** | **WT stressed vs. *CX3CR1 KO* stressed** | **WT non-stressed vs. WT CRS** | ***CX3CR1 KO* non-stressed vs. *CX3CR1 KO CRS*** | **WT non-stressed vs. *CX3CR1 KO* non-stressed** | **WT stressed vs. *CX3CR1 KO* stressed** |
| Mechanical hyperalgesia | | | | | | | | |
| 1. week | 0,0557 | 0,4701 | 0,8764 | 0,0557 | 0,3257 | 0,7091 | 0,9907 | 0,8539 |
| 2. week | <0,0001 | 0,9086 | >0,9999 | <0,0001 | <0,0001 | 0,0009 | >0,9999 | 0,0009 |
| Cold hyperalgesia | | | | | | | | |
| 1. week | <0,0001 | <0,0001 | 0,9004 | 0,0028 | <0,0001 | <0,0001 | 0,1854 | 0,0011 |
| 2. week | <0,0001 | <0,0001 | 0,9889 | 0,001 | <0,0001 | <0,0001 | 0,8541 | 0,8541 |
| S1HL IBA1 - integrated density | 0,0347 | 0,481 | 0,0329 | 0,4799 |  | | | |
| Glia-neuron soma connection | 0,6202 | 0,2097 | <0,0001 | 0,0045 |  |  |  |  |
| PAG- IBA1 integrated density | 0,0435 | 0,9886 | 0,0242 | 0,9019 | 0,2034 | 0,7633 | 0,9999 | 0,5539 |
| CA3-IBA1 integrated density | 0,7062 | 0,5091 | 0,9934 | 0,9007 | 0,6509 | 0,8744 | 0,838 | 0,6059 |
| CeA IBA1-integrated density | 0,9445 | 0,7424 | 0,6829 | 0,9732 | 0,989 | 0,7157 | 0,8122 | 0,9996 |
| PAG- GFAP integrated density | <0,0001 | 0,1941 | 0,8817 | <0,0001 | <0,0001 | 0,9916 | <0,0001 | 0,9895 |
| CA3-GFAP integrated density | 0,6419 | 0,9993 | 0,4645 | 0,918 | 0,0394 | 0,0821 | 0,2422 | 0,3455 |
| Weight change | | | | | | | | |
| 1. week | 0,1433 | 0,0007 | 0,942 | 0,6421 | <0,0001 | <0,0001 | 0,8097 | 0,972 |
| 2. week | 0,2362 | 0,0006 | 0,9575 | 0,4144 | 0,001 | <0,0001 | 0,3707 | 0,9783 |
| Organ weights | | | | | | | | |
| Relative adrenal weight | <0,0001 | <0,0001 | 0,7246 | 0,6916 | <0,0001 | <0,0001 | 0,1551 | 0,9732 |
| Relative thymus weight | 0,1301 | 0,0227 | 0,7404 | 0,2634 | <0,0001 | 0,5208 | 0,3821 | 0,0651 |
| IBA1 | | | | | | | | |
| S1HL cell activation score | 0,0008 | 0,8294 | 0,7453 | 0,0151 | 0,5895 | 0,9991 | 0,9961 | 0,5154 |
| S1HL cell density | 0,8982 | 0,9801 | 0,0797 | 0,2343 | 0,7877 | 0,1221 | 0,0088 | 0,7928 |
| CA3 cell activation score | 0,0007 | 0,9965 | 0,6895 | 0,0035 | 0,0008 | 0,6931 | 0,9195 | 0,0003 |
| CA3 cell density | 0,011 | 0,0148 | 0,0999 | 0,0774 | 0,6273 | 0,3987 | 0,9993 | 0,0951 |
| PAG cell activation score | 0,223 | 0,9592 | 0,8347 | 0,6984 | 0,0632 | 0,9816 | 0,9434 | 0,187 |
| PAG cell density | 0,442 | 0,9533 | 0,0016 | 0,0426 | 0,8767 | 0,4018 | 0,1078 | 0,4668 |
| CeA cell activation score | 0,0896 | 0,0375 | 0,3923 | 0,6525 | 0,7499 | 0,9829 | 0,8728 | 0,5562 |
| CeA cell density | 0,0007 | 0,999 | 0,8405 | <0,0001 | 0,8471 | 0,989 | 0,025 | 0,0569 |
| GFAP | | | | | | | | |
| PAG cell activation score | 0,5334 | 0,4389 | 0,37 | 0,6165 | 0,0135 | 0,4314 | 0,0766 | 0,9133 |
| PAG cell density | 0,7499 | 0,7909 | 0,6526 | 0,8803 | 0,0011 | 0,8862 | 0,8262 | 0,011 |
| CA3 cell activation score | 0,6773 | 0,3245 | 0,1385 | 0,9559 | 0,0209 | 0,9995 | 0,5797 | 0,1547 |
| CA3 cell density | 0,2794 | >0,9999 | 0,6482 | 0,8052 | 0,7414 | 0,9984 | 0,7617 | 0,9957 |

**Supplementary Table 1.** Comprehensive summary of statistical comparisons between experimental groups in wild-type (WT) and CX3CR1 knock-out (KO), non-stressed, chronic restraint stressed (CRS) male and female mice. Reporting the exact p values for all pairwise group comparisons across nociceptive, behavioral, morphological, and immunohistochemical measurements. Statistical analysis was performed with Two-way analysis of variance (ANOVA), followed by Sidak’s test in case of single measurements, and with Two-way repeated measures analysis of variance (RM ANOVA) followed by Tukey’s test for nociceptive and weight measurements, where repeated measurements were performed.

|  | **Vehicle non-stressed vs. vehicle CRS** | ***AZD8797* non-stressed vs.*AZD8797 CRS*** | **Vehicle non-stressed vs. *AZD8797*non-stressed** | **Vehicle stressed vs. *AZD8797* stressed** |
| --- | --- | --- | --- | --- |
| Mechanical hyperalgesia | | | | |
| 1. week | 0,3941 | 0,9987 | 0,9858 | 0,605 |
| 2. week | 0,0792 | 0,9602 | 0,8248 | 0,0132 |
| Cold hyperalgesia | | | | |
| 1. week | <0,0001 | <0,0001 | 0,7494 | 0,9094 |
| 2. week | <0,0001 | <0,0001 | 0,9977 | 0,9926 |
| TST | | | | |
| Immobility time | 0,9487 | 0,8939 | 0,5647 | 0,9555 |
| FST | | | | |
| Immobility time | 0,0028 | 0,2306 | 0,2075 | 0,8075 |
| OFT | | | | |
| Time spent in the periphery | 0,36 | 0,5169 | 0,0175 | 0,8603 |
| Distance moved | 0,999 | 0,3967 | 0,6999 | 0,1199 |
| Time spent moving | 0,3239 | 0,9698 | 0,0169 | 0,354 |
| LDB | | | | |
| Time spent in the light | 0,8476 | 0,447 | 0,5348 | 0,7851 |
| IBA1 | | | | |
| S1HL cell activation score | 0,968 | 0,7844 | 0,9602 | 0,802 |
| S1HL cell density | 0,177 | 0,9898 | 0,8148 | 0,2944 |
| S1HL integrated density | 0,8484 | 0,0821 | 0,2936 | 0,4724 |
| CA3 cell activation score | 0,968 | 0,7844 | 0,9602 | 0,802 |
| CA3 cell density | 0,177 | 0,9898 | 0,8148 | 0,2944 |
| CA3 integrated density | 0,9636 | 0,9899 | 0,7716 | 0,936 |
| PAG cell activation score | 0,2498 | 0,8897 | 0,9945 | 0,1004 |
| PAG cell density | 0,7467 | 0,4537 | 0,7306 | 0,4708 |
| PAG integrated density | 0,6082 | 0,9834 | 0,9898 | 0,5525 |
| CeA cell activation score | 0,6958 | 0,9037 | 0,8218 | 0,7802 |
| CeA cell density | 0,4181 | 0,733 | 0,744 | 0,3746 |
| CeA integrated density | 0,9974 | 0,788 | 0,869 | 0,9998 |
| GFAP | | | | |
| CA3 cell activation score | >0,9999 | >0,9999 | 0,1265 | 0,1673 |
| CA3 cell density | 0,0396 | 0,1644 | 0,951 | 0,7964 |
| CA3 integrated density | 0,7039 | 0,7709 | 0,2618 | 0,9946 |

**Supplementary Table 2.** Comprehensive summary of statistical comparisons between experimental groups in Vehicel-treated and AZD8797-treated, non-stressed, chronic restraint stressed (CRS) male mice. Reporting the exact p values for all pairwise group comparisons across nociceptive, behavioral, morphological, and immunohistochemical measurements. Statistical analysis was performed with Two-way analysis of variance (ANOVA), followed by Sidak’s test in case of single measurements, and with Two-way repeated measures analysis of variance (RM ANOVA) followed by Tukey’s test for nociceptive and weight measurements, where repeated measurements were performed.

|  | **WT non-stressed vs. WT CRS** | ***CX3CR1 KO* non-stressed vs. *CX3CR1 KO CRS*** | **WT non-stressed vs. *CX3CR1 KO* non-stressed** | **WT stressed vs. *CX3CR1 KO* stressed** |
| --- | --- | --- | --- | --- |
| TST | | | | |
| Immobility time | 0,0004 | 0,0153 | 0,9221 | 0,684 |
| FST | | | | |
| Immobility time | 0,4798 | 0,8682 | 0,8916 | 0,4595 |
| OFT | | | | |
| Time spent in the periphery | 0,9918 | 0,8296 | 0,0164 | 0,0415 |
| Distance moved | 0,0776 | 0,0298 | 0,0008 | 0,004 |
| Time spent moving | 0,0347 | 0,0083 | 0,0354 | 0,1525 |
| LDB | | | | |
| Time spent in the light | 0,1112 | 0,9057 | 0,2642 | 0,9957 |

**Supplementary Table 3.** Comprehensive summary of statistical comparisons between experimental groups in wild-type (WT) and CX3CR1 knock-out (KO), non-stressed, chronic restraint stressed (CRS) mice of both sexes, merged. Reporting the exact p values for all pairwise group comparisons in behavioral tests. Statistical analysis was performed with Two-way analysis of variance (ANOVA), followed by Sidak’s test.

|  |  | WT | |  | KO | | |
| --- | --- | --- | --- | --- | --- | --- | --- |
|  |  | ♂ | ♀ | P value | ♂ | ♀ | P value |
| S1HL | Cell activation | 1.075±0.01747 | 1.127±0.01360 | 0,1335 | 1.1255±0.01495 | 1.124±0.02482 | 0,4077 |
|  | Cell density | 406.5±26.21 | 452.5±12.74 | 0,1931 | 357.3±11.19 | 388.4±2.600 | 0,4251 |
| PAG | Cell activation | 1.102±0.02397 | 1.126±0.01609 | 0,0707 | 1.1255±0.02710 | 1.141±0.02872 | 0,4634 |
|  | Cell density | 264.3±6.077 | 292.7±5.467 | 0,8961 | 224.8±4.519 | 271.4±4.870 | >0,9999 |
| CA3 | Cell activation | 1.069±0.02447 | 1.146±0.01868 | 0,1481 | 1.134±0.02676 | 1.158±0.008889 | 0,7888 |
|  | Cell density | 377.3±12.06 | 420.1±6.898 | 0,1001 | 348.5±8.102 | 419.5±11.32 | >0,9999 |
| CeA | Cell activation | 1.103±0.01267 | 1.128±0.01791 | 0,7707 | 1.057±0.009485 | 1.115±0.01199 | 0,8723 |
|  | Cell density | 438.3±30.31 | 442.5±7.629 | 0,2822 | 394.5±6.327 | 394.5±6.327 | 0,8961 |

**Supplementary Table 4.** Microglia cell activation (1-5) and cell density (cell/mm2) of the indicated groups of wild type (WT) and CX3CR1 knock-out (KO) animals, presented as mean±SEM in the in the somatosensory cortex (S1HL), periaqueductal gray matter (PAG), hippocampus Cornu Ammonis 3 (CA3), central amygdala (CeA). P values indicate comparisons between male and female animals within the same genotype.

|  |  | WT | | | KO | | |
| --- | --- | --- | --- | --- | --- | --- | --- |
|  |  | ♂ | ♀ | P value | ♂ | ♀ | P value |
| PAG | Cell activation | 1.307±0.1007 | 1.084±0.03818 | 0,0617 | 1.146±0.07574 | 1.264±0.05600 | 0,9774 |
|  | Cell density | 158.5±57.77 | 74.10±20.85 | 0,6289 | 111.1±36.49 | 92.11±7.543 | 0,5485 |
| CA3 | Cell activation | 1.665±0.1339 | 1.308±0.04665 | 0,0707 | 1.139±0.09212 | 1.428±0.1376 | 0,4634 |
|  | Cell density | 495.9±59.00 | 443.4±30.24 | 0,2018 | 545.2±34.34 | 482.1±40.43 | 0,9221 |

**Supplementary Table 5.** Astrocyte cell activation (1-5) and cell density (cell/mm^2^) of the indicated groups of wild type (WT) and CX3CR1 knock-out (KO) animals, presented as mean±SEM in the in the somatosensory cortex (S1HL), periaqueductal gray matter (PAG), hippocampus Cornu Ammonis 3 (CA3), central amygdala (CeA). P values indicate comparisons between male and female animals within the same genotype.

| Multiple comparisons | F values |
| --- | --- |
| Mechanical hyperalgesia ♂ (Fig. 1. B) | interaction: F (3, 75) = 6,437; P=0,0006 |
| Mechanical hyperalgesia ♀ (Fig. 1. D) | interaction: F (3, 72) = 3,410; P=0,0220 |
| Cold hyperalgesia ♂ (Fig. 1. F) | interaction: F (3, 72) = 420,0; P<0,0001 |
| Cold hyperalgesia ♀ (Fig. 1. H) | interaction: F (3, 72) = 3,637; P=0,0167 |
| S1HL IBA1 - Integratwed density ♂ (Fig . 2. A) | interaction: F (1, 43) = 6,516; P=0,0143 |
| Glia/neuron soma connection (%) (Fig. 2. A) | genotype factor: F (1, 356) = 37,18; P<0,0001 |
| PAG IBA1 - Integratwed density ♂ (Fig . 3. A) | interaction: F (1, 20) = 5,012; P=0,0367 |
| PAG GFAP - Integratwed density ♂ (Fig . 4. A) | interaction: F (1, 20) = 29,09; P<0,0001 |
| PAG GFAP - Integratwed density ♀ (Fig . 4. B) | interaction: F (1, 20) = 21,77; P=0,0001 |
| CA3 GFAP - Integratwed density ♀ (Fig . 4. B) | stress factor: F (1, 18) = 11,26; P=0,0035 |
| Mechanical hyperalgesia (%) (Fig. 5. A) | stress factor: F (3, 29) = 3,111; P=0,0416 |
| Cold hyperalgesia (%) (Fig. 5. B) | stress factor: F (3, 29) = 26,14; P<0,0001 |
| Behavioural tests | |
| Time spent immobile in TST (Suppl. Fig. 1. A) | stress factor: F (1, 82) = 21,84; P<0,0001 |
| Time spent in the periphery in OFT (Suppl. Fig. 1. C) | genotype factor: F (1, 80) = 16,77; P=0,0001 |
| Distance moved in OFT (Suppl. Fig. 1. D) | stress factor: F (1, 85) = 10,48; P=0,0017 genotype factor: F (1, 85) = 23,71; P<0,0001 |
| Time spent moving in OFT (Suppl. Fig. 1. E) | stress factor: F (1, 85) = 14,46; P=0,0003 genotype factor: F (1, 85) = 8,766; P=0,0040 |
| Weight change ♂ (Suppl. Fig. 2. C) | stress factor: F (3, 33) = 24,52; P<0,0001 |
| Weight change ♀ (Suppl. Fig. 2. C) | stress factor: F (1, 68) = 51,16; P<0,0001 |
| Relative tymus weight ♂ (Suppl. Fig . 2. E) | stress factor: F (3, 150) = 13,43; P<0,0003 |
| Relative tymus weight ♀ (Suppl. Fig . 2. F) | stress factor: F (1, 33) = 61,64; P<0,0001 |
| Relative adrenal gland weight ♂ (Suppl. Fig . 2. G) | stress factor: F (1, 32) = 15,97; P=0,0004 |
| Relative adrenal gland weight ♀ (Suppl. Fig . 2. H) | stress factor: F (1, 70) = 9,928; P=0,0025 |
| IBA1 | |
| S1HL cell activation score ♂ (Suppl. Fig . 3. A) | interaction: F (1, 42) = 6,069; P=0,0179 |
| S1HL cell density ♀ (Suppl. Fig . 3. D) | genotype factor: F (1, 20) = 7,317; P=0,0136 |
| PAG cell density ♂ (Suppl. Fig . 3. F) | genotype factor: F (1, 20) = 20,75; P=0,0002 |
| CA3 cell activation score ♂ (Fig . 3. I) | interaction: F (1, 19) = 9,458; P=0,0062 |
| CA3 cell density ♂ (Suppl. Fig . 3. J) | stress factor: F (1, 20) = 9,145; P=0,0067 |
| CA3 cell activation score ♀ (Fig . 3. K) | interaction: F (1, 20) = 12,71; P=0,0019 |
| CeA cell density ♂ (Suppl. Fig . 3. N) | interaction: F (1, 19) = 12,72; P=0,0021 |
| CeA cell density ♀ (Suppl. Fig . 3. P) | genotype factor: F (1, 20) = 12,98; P=0,0018 |
| GFAP | |
| PAG activation score ♀ (Suppl. Fig . 4. C) | stress factor: F (1, 20) = 8,886; P=0,0074 |
| PAG cell density ♀ (Suppl. Fig . 4. D) | interaction: F (1, 20) = 6,731; P=0,0173 |
| Behavioural tests with | |
| Time spent immobile in FST (Suppl. Fig. 5. B) | stress factor: F (1, 25) = 13,98; P=0,0010 |
| Time spent in the periphery in OFT (Suppl. Fig. 5. C) | genotype factor: F (1, 30) = 5,553; P=0,0252 |
| Time spent moving in OFT (Suppl. Fig. 5. E) | genotype factor: F (1, 29) = 8,800; P=0,0060 |

**Supplemetary Table 6.** All F values corresponding to statistically significant effects from all performed analyses.
